# Parameter Decoupling and Standardization of an RDE Protocol for CO Tolerance Evaluation of the Alkaline Hydrogen Oxidation Reaction

**DOI:** 10.3390/ma19163460

**Published:** 2026-08-14

**Authors:** Hong-Fei Xing, Mei-Li Wang, Hao-Ran Wu, Wei-Dong Li, Bang-An Lu

**Affiliations:** College of Materials Science and Engineering, Zhengzhou University, Zhengzhou 450001, China

**Keywords:** CO tolerance, alkaline hydrogen oxidation reaction, rotating disk electrode, catalyst loading, electrochemical impedance spectroscopy

## Abstract

CO poisoning remains a major challenge for hydrogen fuel cells, and rotating disk electrode (RDE) measurements are widely used for preliminary screening of CO-tolerant catalysts. However, variations in key testing parameters, including catalyst loading, CO exposure time, and linear sweep voltammetry (LSV) scan rate, can substantially affect the apparent CO tolerance response and compromise cross-study comparability. Here, Pt/C was employed as a model catalyst to systematically decouple the effects of these parameters on CO tolerance evaluation of the hydrogen oxidation reaction (HOR). Catalyst loading was identified as a critical factor: at a low Pt loading of 5 μg_Pt_ cm^−2^, CO adsorption caused a severe decrease in the HOR limiting current, whereas at a high loading of 40 μg_Pt_ cm^−2^, excess available Pt sites markedly diluted the apparent poisoning effect. Electrochemical impedance analysis further revealed distinct loading-dependent changes in interfacial charge-transfer and mass-transport processes under CO-containing conditions. In addition, prolonged CO pre-exposure increased surface poisoning and current loss, while slower LSV scanning amplified the apparent poisoning response by extending the effective CO exposure time. Based on these findings, we propose a standardized RDE evaluation principle based on low catalyst loading, sufficient CO pre-exposure, and slow potential scanning, providing a more reproducible and comparable benchmark for evaluating catalyst-dependent resistance to CO poisoning under controlled RDE conditions.

## 1. Introduction

Anion exchange membrane fuel cells (AEMFCs) are regarded as promising next-generation energy conversion technologies because their alkaline operating environment offers the potential to reduce reliance on platinum group metal (PGM) catalysts [1,2]. However, compared with the rapid hydrogen oxidation reaction (HOR) kinetics of Pt in acidic media, the alkaline HOR is approximately two orders of magnitude slower and has become a major bottleneck limiting further performance improvements in AEMFCs [3,4]. Accordingly, extensive efforts have been devoted to the development of noble-metal-based catalysts, including Pt-, Ru-, Pd-, and Rh-based systems. Strategies such as alloying, electronic structure modulation, construction of oxophilic sites, and metal–support interfacial engineering have been widely explored to regulate H- and OH-related adsorption processes as well as interfacial water structures, thereby enhancing alkaline HOR activity [5,6,7,8,9].

Beyond intrinsic activity, the durability of HOR catalysts under realistic operating conditions is equally critical for practical application, wherein CO poisoning represents a primary obstacle. Practical hydrogen feeds, particularly those derived from reforming processes, often contain trace amounts of CO impurities. Through strong σ-donation and π-back-donation interactions, CO adsorbs strongly on platinum-group-metal surfaces and occupies active sites required for H_2_ adsorption and dissociation; even ppm-level CO can cause severe deactivation of anode catalysts [10,11]. Therefore, developing HOR catalysts that simultaneously exhibit high intrinsic activity and strong CO tolerance has become an important research objective. Current strategies mainly include weakening CO adsorption through electronic modulation, introducing oxophilic sites to promote oxidative CO removal, and constructing bifunctional interfaces that facilitate CO oxidation and desorption [12,13,14,15].

Among the available approaches for evaluating CO tolerance, membrane electrode assembly (MEA) testing provides the most direct assessment of catalyst poisoning behavior under practical fuel-cell operating conditions. However, MEA testing is inherently limited by operational complexity, long testing periods, and high experimental costs. A typical MEA-based CO tolerance evaluation requires preparing at least nine nominally identical samples from the same batch to perform measurements at no fewer than three different current densities, ensuring statistical reliability. Each testing sequence comprises three stages—steady-state operation, poisoning, and recovery—with the steady-state stage maintained for at least 60 min [16]. In addition, MEA fabrication involves multiple processing steps, and batch-to-batch variations are difficult to eliminate completely, thereby presenting a substantial barrier to high-throughput catalyst screening. In contrast, the rotating disk electrode (RDE) technique, owing to its operational simplicity, short experimental duration, and good reproducibility, has become a prevalent tool for the preliminary screening and mechanistic study of HOR catalysts [17,18,19]. Through controlled electrode rotation, the RDE configuration provides well-defined mass-transport conditions and requires only microgram-scale quantities of catalyst, allowing individual measurements to be completed within several hours. These characteristics make the RDE technique particularly advantageous for rapid catalyst screening, for which MEA testing is considerably less practical [17,18].

Despite the widespread use of the RDE technique, a unified protocol for CO tolerance evaluation has not yet been established. Two approaches are commonly employed in the literature. The first evaluates CO tolerance by comparing linear sweep voltammetry (LSV) polarization curves recorded in CO-containing H_2_ with those obtained in pure H_2_, whereas the second monitors current decay through chronoamperometric current–time (i–t) measurements at a fixed potential under a CO-containing atmosphere. These two methods provide complementary but distinct information. The i–t method records the time-dependent current decay at a fixed potential and thus directly evaluates poisoning stability under a specific operating condition. In contrast, LSV provides a complete polarization response over a broad potential window following CO exposure, yielding a potential-dependent map of residual activity that can reveal the differential influence of CO poisoning on HOR kinetics across different potential regions. A key advantage of the LSV-based protocol, which makes it particularly suited for this work, lies in its ability to temporally decouple CO poisoning from the activity readout: the catalyst first undergoes controlled CO exposure under predefined conditions, and its residual activity is assessed during a subsequent potential scan. This decoupling enables systematic investigation of individual parameters such as poisoning time, scan rate, and catalyst loading, with the scan rate itself becoming an experimental variable. For this reason, LSV was selected as the primary analytical method in this work to deconvolute the individual effects of key testing parameters.

In this work, it is important to distinguish the catalyst-intrinsic resistance to CO poisoning from the experimentally observed apparent CO tolerance response. The former refers to the material-dependent propensity to resist CO-induced deactivation under otherwise identical electrochemical conditions and is governed by physicochemical factors such as CO adsorption strength and kinetics, competitive adsorption of H- and OH-related species, CO oxidation/desorption behavior, surface electronic structure, and local interfacial configuration. In contrast, experimentally measured activity retention reflects the combined influence of these catalyst-intrinsic properties and the specific testing conditions.

Accordingly, the apparent CO tolerance response obtained from LSV measurements is not determined solely by the catalyst-intrinsic resistance to CO poisoning, but is also affected by experimental parameters including noble-metal loading, CO concentration, scan rate, pre-exposure time, and holding potential [17,18,19,20]. Considerable variations in CO tolerance evaluation conditions have been reported for HOR catalysts, which may alter the surface CO coverage and the number of remaining available active sites, thereby compromising the comparability of results obtained across different studies. In particular, LSV limiting-current retention is intrinsically an apparent metric that is closely coupled to both the measurement timescale and electrode configuration. CO adsorption may already occur during the constant-potential resting period before the LSV scan begins, while the subsequent scan rate further determines the effective exposure time under the CO-containing atmosphere. In addition, catalyst loading determines the total number of available active sites. Excess active sites at high loading may dilute the apparent impact of CO poisoning, whereas low loading can amplify the influence of CO coverage on the limiting current. Therefore, LSV current retention measured under CO-containing conditions should be regarded as a protocol-dependent apparent metric rather than a direct or absolute measure of the catalyst-intrinsic resistance to CO poisoning.

Motivated by these considerations, this work employs structurally well-defined Pt/C as a model catalyst to systematically investigate the influence of key RDE testing parameters on the apparent CO tolerance evaluation of alkaline HOR catalysts. Using a single-variable-at-a-time approach, we deconvolute the effects of pre-scan holding time, holding potential, scan rate, and noble-metal loading on limiting-current decay and CO coverage. Electrochemical impedance spectroscopy (EIS) combined with distribution of relaxation times (DRT) analysis is further employed to elucidate how catalyst loading influences the interfacial kinetic processes associated with CO poisoning. Based on these mechanistic insights, we propose a standardized evaluation protocol aimed at improving the reliability and cross-study comparability of RDE-based CO tolerance measurements.

## 2. Materials and Methods

### 2.1. Synthesis of Pt/C Catalysts

KOH (43 mg) was dissolved in 20 mL of ethylene glycol under ultrasonication, followed by magnetic stirring. Subsequently, 500 μL of a 40 mg mL^−1^ H_2_PtCl_6_·6H_2_O solution was added, and the mixture was stirred for 10 min to ensure homogeneous dispersion. The resulting solution was then subjected to microwave heating under gentle stirring. After the temperature reached 160 °C, it was maintained for 3 min, after which the reaction mixture was removed and rapidly cooled in an ice-water bath. An equal volume of 1 M HCl solution was then added to the reaction mixture. After standing for 10 min, the product was collected by centrifugation and washed three times with 1 M HCl solution. Subsequently, 5 mg of the obtained Pt nanoparticles were dispersed in ethanol and mixed with 20 mg of conductive carbon black, followed by ultrasonication for 2 h. The resulting suspension was filtered using ultrapure water and vacuum-dried at 80 °C for 12 h to obtain the nominal 20 wt% Pt/C catalyst.

Pt/C catalysts with Pt mass fractions of 8, 12, and 16 wt% were prepared by dispersing the above Pt/C product in ethanol and mixing it with appropriate amounts of conductive carbon black.

### 2.2. Electrochemical Measurements

Electrochemical measurements were performed in a conventional three-electrode cell using a CHI 760E electrochemical workstation (Shanghai Chenhua Instrument Co., Ltd., Shanghai, China). A graphite rod with a diameter of 5 mm, a saturated calomel electrode (SCE), and a glassy carbon rotating disk electrode (GCE-RDE) were used as the counter, reference, and working electrodes, respectively. Before each measurement, the RDE was polished with 50 nm Al_2_O_3_ slurry and thoroughly rinsed with deionized water. All potentials were converted to the reversible hydrogen electrode (RHE) scale.

The catalyst ink was prepared by dispersing 2 mg of catalyst in 0.5 mL of an isopropanol/water mixture (*v*/*v* = 3:1), and then 5 μL of a dispersion solution containing 5% Nafion was added, followed by ultrasonication to obtain a homogeneous suspension. An appropriate amount of the catalyst ink was drop-cast onto the RDE to achieve a Pt loading of 20 μg_Pt_ cm^−2^. HOR measurements were conducted in H_2_-saturated 0.1 M KOH electrolyte at a rotation rate of 1600 rpm and a scan rate of 5 mV s^−1^. All HOR polarization curves were collected using a positive-going LSV scan direction (from low potential to high potential). Control measurements of the bare carbon support were conducted under the same electrochemical conditions as those used for Pt/C.

The maximum current is reduced by calculating according to the following equation:

(1) $$∆I_{lim}=\frac{i_{CO/H_{2}}}{i_{H_{2}}}$$

$i_{H_{2}}$ represents the maximum current in the pure H_2_ before poisoning, while $i_{CO/H_{2}}$ represents the maximum current in the 1000 ppm CO/H_2_ after poisoning. Both the maximum currents are taken at 200 mV vs. RHE.

The fraction of sites remaining accessible to hydrogen under CO-containing conditions was determined from the ratio *Q*_H,CO_/*Q*_H,clean_, and the CO-blocked fraction (*θ*_CO_) was calculated according to Equation (2) [21,22]:

(2) $$\theta_{CO}=1-\frac{Q_{H,CO}}{Q_{H,clean}}$$

where *Q_H,clean_* is the integrated H_upd_ desorption charge of the clean Pt surface and *Q_H,CO_* is the residual H_upd_ desorption charge after CO exposure. Accordingly, *Q*_H,CO_/*Q*_H,clean_ represents the fraction of Pt sites remaining accessible to hydrogen adsorption, whereas *θ*_CO_ represents the complementary fraction rendered unavailable by CO adsorption.

The turnover frequency (TOF) was calculated according to the following equation:

(3) $$TOF=\frac{i_{k}}{nFn_{active}}$$

where *i*_k_ is the kinetic current (A), *n* represents the number of electrons transferred per reaction molecule, *F* is the Faraday constant (96,485 C mol^−1^), and *n*_active_ stands for the molar amount of active sites of the catalyst.

Unless otherwise specified, quantitative electrochemical measurements were independently repeated using at least five separately prepared electrodes, and the corresponding statistical data are presented as the mean ± standard deviation (SD). Representative polarization, cyclic voltammetry, chronoamperometric, and impedance curves are shown for clarity.

## 3. Results and Discussion

### 3.1. Structural Characterization

**Figure 1:**
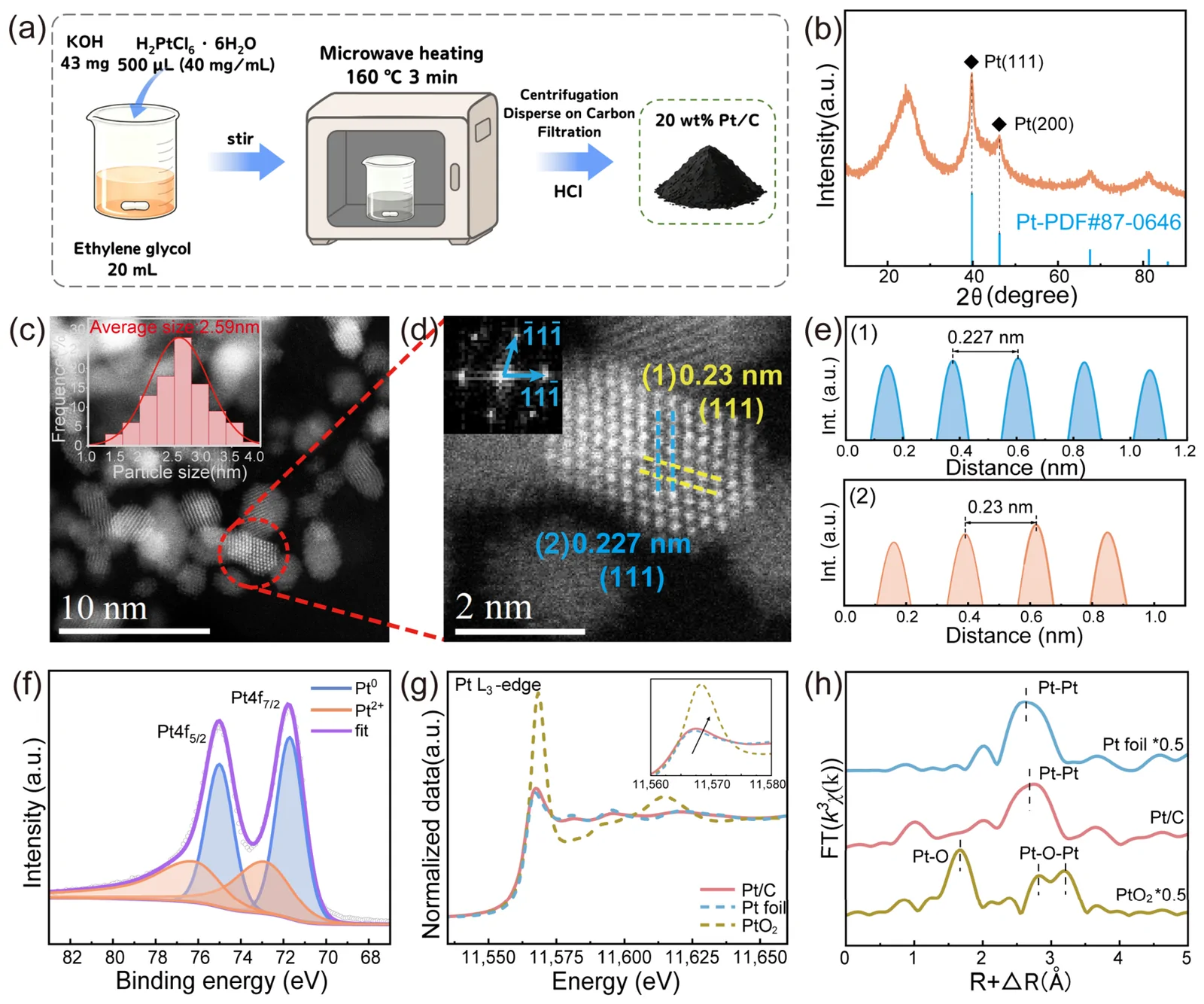
Synthesis and structural characterization of the Pt/C catalyst. (**a**) Schematic illustration of the preparation of Pt/C by the microwave-assisted ethylene glycol reduction method; (**b**) X-ray diffraction (XRD) pattern of Pt/C; (**c**,**d**) High-angle annular dark-field scanning transmission electron microscopy (HAADF-STEM) images of Pt/C, with the inset in (**c**) showing the particle-size distribution and the inset in (**d**) showing the corresponding fast Fourier transform (FFT) diffraction pattern; (**e**) atomic intensity profiles along directions (1) and (2) marked in (**d**); (**f**) high-resolution Pt 4f X-ray photoelectron spectroscopy (XPS) spectrum of Pt/C; (**g**) Pt L_3_-edge X-ray absorption near-edge structure (XANES) spectra; (**h**) k^3^-weighted Fourier-transformed extended X-ray absorption fine structure (FT-EXAFS) spectra of Pt.

Figure 1a illustrates the synthesis of the Pt/C catalyst via a microwave-assisted ethylene glycol reduction method. In this approach, ethylene glycol serves simultaneously as the solvent and reducing agent, while rapid and uniform microwave heating under alkaline conditions promotes the reduction of the Pt precursor and the formation of Pt nanoparticles supported on carbon. The X-ray diffraction (XRD) pattern in Figure 1b exhibits two prominent diffraction peaks at approximately 39.8° and 46.2°, which can be assigned to the (111) and (200) planes of face-centered cubic Pt, respectively, indicating the good crystallinity of the obtained Pt nanoparticles. Inductively coupled plasma atomic emission spectroscopy (ICP-AES) further confirmed an actual Pt loading of approximately 18.5 wt% (Table S1), in good agreement with the nominal value.

Transmission electron microscopy (TEM) and aberration-corrected high-angle annular dark-field scanning transmission electron microscopy (HAADF-STEM) revealed that the Pt nanoparticles were uniformly dispersed on the carbon support without obvious aggregation (Figure 1c and Figure S1a,b). Particle-size analysis gave an average Pt nanoparticle diameter of approximately 2.6 nm. Higher-resolution HAADF-STEM images further revealed well-resolved lattice fringes and ordered atomic arrangements (Figure 1d and Figure S1c). The corresponding fast Fourier transform (FFT) pattern was consistent with the diffraction pattern expected for fcc Pt viewed along the [011] zone axis. The interplanar spacings measured along the two directions marked in Figure 1e were approximately 0.227 and 0.230 nm, both corresponding to the Pt (111) planes and consistent with the XRD results.

The oxidation state and local coordination environment of Pt/C were further investigated by X-ray photoelectron spectroscopy (XPS) and X-ray absorption fine structure spectroscopy (XAFS). As shown in Figure 1f and Figure S2, the Pt 4f spectrum was dominated by metallic Pt, with only a small fraction of oxidized Pt species attributable to surface oxidation. In the Pt L_3_-edge X-ray absorption near-edge structure (XANES) spectrum, the white-line intensity of Pt/C was close to that of Pt foil (Figure 1g), indicating that Pt predominantly existed in the metallic state. The k^3^-weighted Fourier-transformed extended X-ray absorption fine structure (FT-EXAFS) spectrum further showed that Pt–Pt coordination dominated in Pt/C, with no obvious Pt–O coordination peak observed (Figure 1h). These results confirm the successful preparation of a Pt/C catalyst with a well-defined Pt loading, uniform particle size, homogeneous dispersion, and predominantly metallic Pt species, providing a suitable model catalyst for the systematic investigation of testing-parameter effects on CO tolerance evaluation. Carbon black was selected as a conventional conductive support to construct a reproducible Pt/C model catalyst. Control electrochemical measurements of the bare carbon support showed negligible HOR activity in H_2_ and no discernible H_upd_ or CO adsorption/stripping features under the present conditions (Figure S3), confirming that the HOR response and CO-poisoning behavior discussed here originate predominantly from the Pt sites rather than from the carbon support.

### 3.2. Protocol Dependence of the Apparent CO Tolerance of Pt/C

Prior to investigating the protocol-dependent CO tolerance behavior, the intrinsic HOR activity of the prepared Pt/C catalyst was evaluated. The Pt/C catalyst exhibited a specific activity (SA) of 1.15 mA cm_ECSA_^−2^ and a mass activity (MA) of 0.48 A mg_Pt_^−1^. The corresponding turnover frequency (TOF) was also calculated and is provided in Figure S4. Compared with other literature [23,24,25], these activity metrics fall within the range reported for conventional Pt/C benchmarks, confirming that the prepared Pt/C catalyst represents a reliable model system for subsequent CO tolerance investigations. After establishing a structurally well-defined and uniformly dispersed Pt/C model catalyst, we first investigated its HOR response in H_2_ containing 1000 ppm CO. At present, two approaches are commonly used in the literature to evaluate the CO tolerance of HOR catalysts: comparison of LSV polarization curves recorded in pure H_2_ and CO/H_2_ atmospheres, and chronoamperometric measurements at a fixed potential under a CO-containing atmosphere [26]. However, our measurements revealed that these two approaches yielded markedly different apparent CO tolerance results for the same Pt/C catalyst.

**Figure 2:**
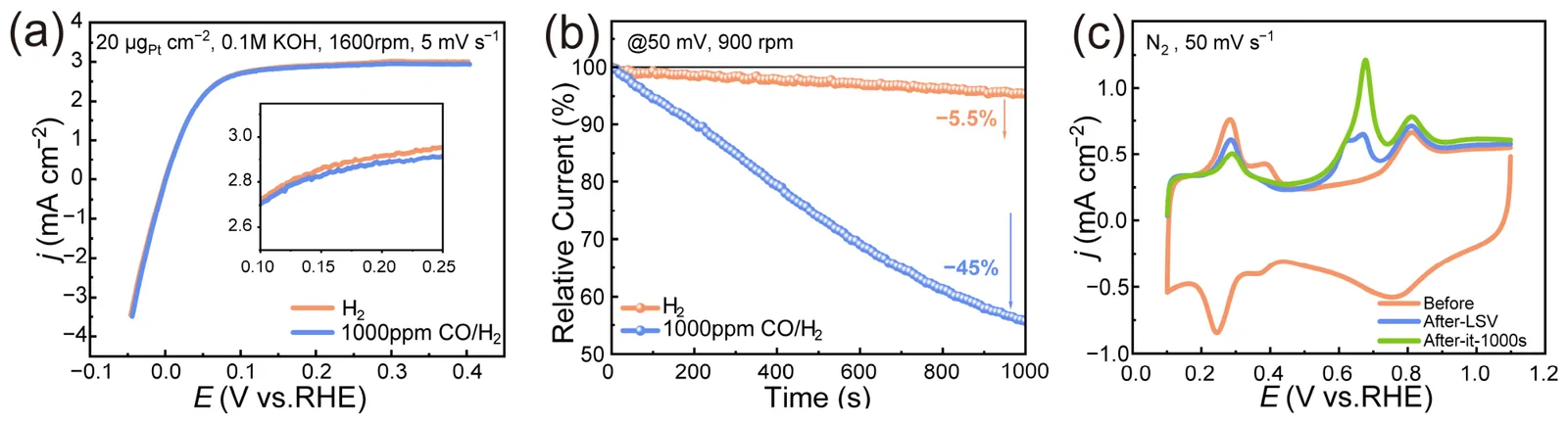
Protocol-dependent apparent CO tolerance of the Pt/C catalyst. (**a**) Comparison of linear sweep voltammetry (LSV) polarization curves measured at a Pt loading of 20 μg_Pt_ cm^−2^ in H_2_ and 1000 ppm CO/H_2_ atmospheres; (**b**) comparison of current-time (i–t) responses measured at a Pt loading of 20 μg_Pt_ cm^−2^ in H_2_ and 1000 ppm CO/H_2_ atmospheres; (**c**) corresponding CO stripping cyclic voltammograms recorded after the tests shown in (**a**,**b**).

As shown in Figure 2a, at a Pt loading of 20 μg_Pt_ cm^−2^, the limiting current of Pt/C in 1000 ppm CO/H_2_ showed almost no decrease relative to that measured in pure H_2_. Control experiments confirmed that the slight dilution of H_2_ caused by the introduction of 1000 ppm CO had a negligible effect on the limiting current (see Section S1.2 of the Supplementary Materials). Based solely on the LSV results, the Pt/C catalyst would therefore appear to exhibit excellent apparent CO tolerance. In sharp contrast, the chronoamperometric measurements revealed substantially more severe poisoning behavior. When the electrode was held at 50 mV for 1000 s, the current decreased by only approximately 5.5% in pure H_2_, whereas a decay of approximately 45% was observed in 1000 ppm CO/H_2_ (Figure 2b). This pronounced difference indicates that CO continuously accumulates on the Pt surface during constant-potential exposure, progressively blocking available active sites and suppressing the HOR [27].

To further clarify the origin of the discrepancy between the two evaluation methods, the electrodes were characterized by cyclic voltammetry (CV) after the respective tests. As shown in Figure 2c, Pt/C subjected to a 1000 s constant-potential hold in 1000 ppm CO/H_2_ exhibited a substantially larger CO stripping peak area than the sample tested by direct LSV in CO/H_2_, indicating a markedly higher surface CO coverage [28]. This result reveals the fundamental origin of the different outcomes obtained from the two testing protocols. During direct LSV measurements, the effective exposure time of the electrode to CO/H_2_ is relatively short, and CO adsorption has not reached a substantial coverage level, resulting in only a minor decrease in the limiting current. In contrast, constant-potential holding substantially prolongs the period available for CO accumulation on the Pt surface, leading to more severe activity decay.

These observations demonstrate that the experimentally measured CO tolerance response in an RDE configuration is determined jointly by catalyst-intrinsic physicochemical properties and the testing protocol. Therefore, LSV current retention under a CO-containing atmosphere should be interpreted as an apparent CO tolerance metric rather than a direct measure of the catalyst-intrinsic resistance to CO poisoning. Motivated by this finding, we further surveyed the CO tolerance evaluation conditions reported for HOR catalysts [29,30,31]. As summarized in Table 1, substantial variations exist among different studies in several key parameters, including noble-metal loading, CO concentration, LSV scan rate, holding potential, and poisoning duration. The principal outcome of this survey is therefore not a direct ranking of the CO tolerance performance of the reported catalysts, but rather the recognition that these values were obtained under markedly different testing conditions and, consequently, may correspond to different surface poisoning states. Such protocol heterogeneity complicates direct comparison of activity-retention values across catalyst systems. Accordingly, Pt/C was subsequently employed as a model catalyst in single-variable control experiments to systematically decouple the effects of CO pre-exposure conditions, LSV scan rate, and catalyst loading on the apparent CO tolerance response of alkaline HOR catalysts.

### 3.3. Effect of Electrochemical Testing Parameters on Apparent CO Tolerance Evaluation

After confirming that different testing protocols can lead to different apparent CO tolerance results for the same Pt/C catalyst, we further examined the effect of the constant-potential resting period before the LSV measurement. In practical electrochemical testing, after the working electrode is connected to the three-electrode system and the initial potential is set, the electrode is generally held at that potential for a certain period before the LSV scan begins. For HOR measurements in CO/H_2_, this period should not be regarded merely as a procedurally inactive equilibration period, because CO adsorption and accumulation on Pt may already occur during this stage. As a result, the initial poisoning state of the electrode surface can be altered before the polarization scan begins, thereby affecting the apparent CO tolerance reflected by the subsequent LSV response [48].

**Figure 3:**
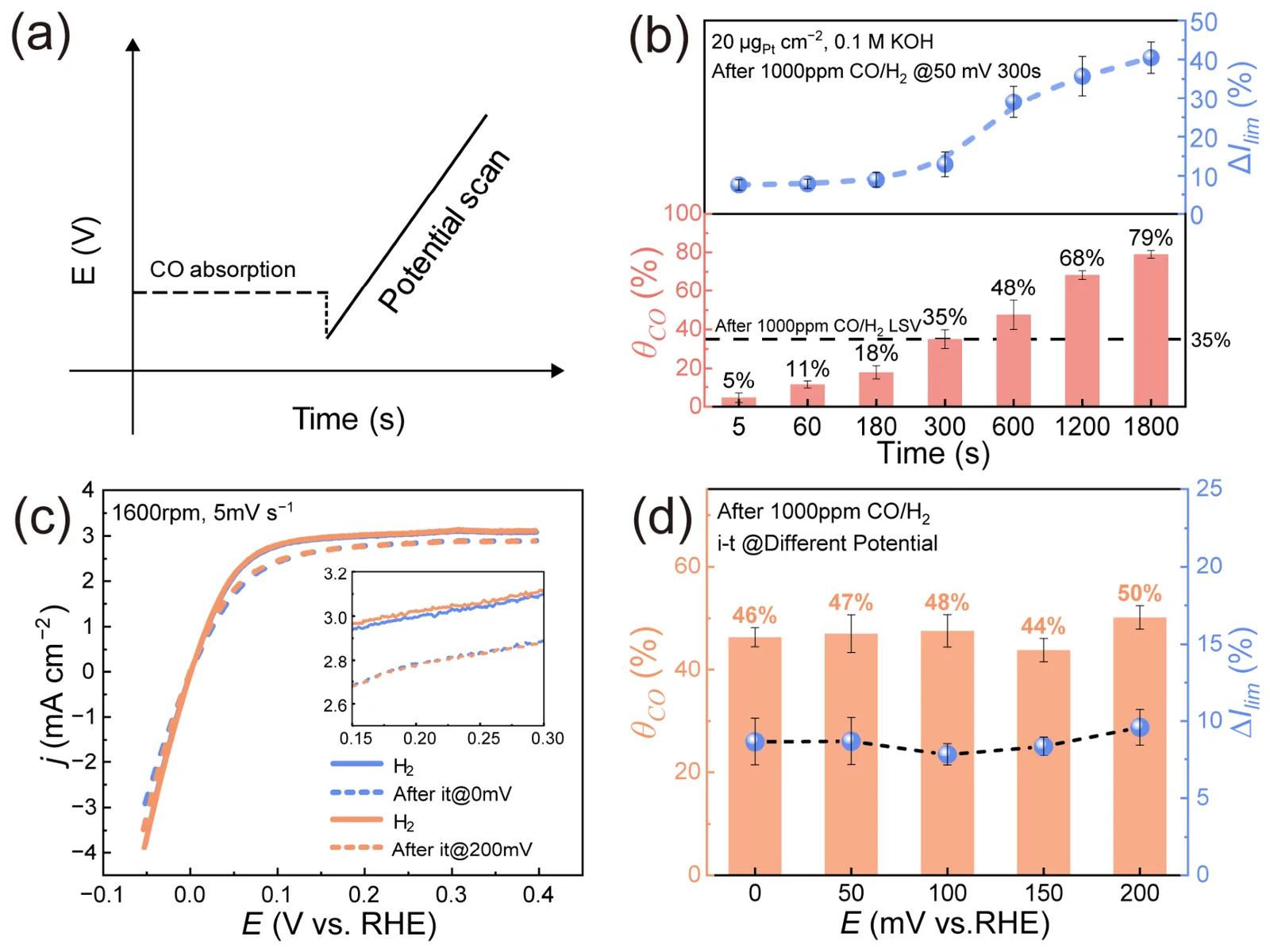
Effects of resting time and holding potential on CO tolerance evaluation. (**a**) Schematic illustration of the practical testing sequence, which typically includes a constant-potential resting period followed by a potential-sweep polarization measurement; (**b**) variations in LSV limiting-current decay and CO coverage after different constant-potential holding times; (**c**) comparison of LSV polarization curves after 300 s constant-potential holds at 0 and 200 mV; (**d**) variations in LSV limiting-current decay and CO coverage after 300 s holds at different potentials. Data points represent the mean of at least five independent trials conducted under identical conditions; error bars denote SDs.

Figure 3a schematically illustrates the potential program before and during the LSV measurement under a CO-containing atmosphere. Prior to the potential scan, the electrode is first held at a prescribed potential, during which CO can progressively adsorb on Pt active sites. The potential is then swept linearly, initiating the dynamic LSV measurement. Consequently, the resulting CO/H_2_-LSV curve reflects the combined effects of constant-potential pre-exposure and subsequent potential scanning. To clarify the influence of resting time, the Pt loading was fixed at 20 μg_Pt_ cm^−2^, while the CO concentration, holding potential, and LSV scan rate were kept constant. Only the constant-potential resting time in 1000 ppm CO/H_2_ was varied, and the resulting limiting-current decay and post-test CO coverage were compared.

As shown in Figure 3b and Figure S5, when the resting time was short, the LSV limiting current measured in 1000 ppm CO/H_2_ differed only slightly from that obtained in pure H_2_, indicating that short-term CO exposure had not yet caused severe blockage of Pt active sites. As the constant-potential resting time increased, the limiting-current decay became progressively more pronounced. After resting at 50 mV for approximately 300 s, a more evident decrease in the limiting current was observed, while the CO coverage determined by CV reached approximately 30%. Further extension of the resting time resulted in a continued increase in CO coverage accompanied by a further decrease in the LSV limiting current. The similar trends of these two parameters indicate that CO accumulation on the Pt surface during the resting period is a major origin of the subsequent HOR activity loss [27]. These results demonstrate that the pre-LSV resting time in a CO-containing atmosphere can substantially alter the poisoning state of the catalyst surface and thereby affect the apparent CO tolerance obtained from the subsequent LSV measurement. Accordingly, unless otherwise specified, the subsequent LSV measurements were performed after a 300 s constant-potential hold at 50 mV.

In addition to resting time, the holding potential itself may also influence the CO adsorption state. Therefore, we further examined the effect of different constant potentials on the subsequent LSV response. As shown in Figure 3c, after a 300 s hold at overpotentials of 0 and 200 mV, the LSV limiting current of Pt/C in 1000 ppm CO/H_2_ decreased relative to that in pure H_2_, but the results obtained at the two holding potentials were similar. A broader comparison over the 0–200 mV overpotential range is presented in Figure 3d and Figures S6 and S7. After treatment at different holding potentials, the CO coverage of Pt/C remained within a relatively narrow range of approximately 45–50%, while the corresponding limiting-current decay also varied only slightly and exhibited no clear potential dependence. These results indicate that, within the low-overpotential range examined in this work, the degree of CO poisoning is governed primarily by the exposure time in the CO-containing atmosphere, whereas changes in the holding potential have a comparatively limited influence on CO accumulation and the subsequent decay in LSV limiting current. It should be noted that the CO coverage here was measured after potentiostatic holding followed by LSV testing, and thus the obtained CO coverage is higher than the data acquired at different potentiostatic durations in Figure 3b.

Overall, the results in Figure 3 demonstrate that the constant-potential resting period before LSV measurement can significantly influence the CO tolerance evaluation of HOR catalysts, with resting time being more critical than holding potential. Longer resting times increase the CO coverage on Pt and lead to a more pronounced decrease in the subsequent LSV limiting current, whereas varying the holding potential within the 0–200 mV overpotential range has only a minor effect on either CO coverage or limiting-current decay. Therefore, the pre-LSV resting time should be explicitly reported and strictly controlled in CO tolerance measurements; otherwise, apparent CO tolerance results obtained from different experiments may not be directly comparable.

After establishing that the constant-potential resting period before LSV measurement affects CO accumulation, we further investigated the influence of LSV scan rate on the apparent CO tolerance response. Like the constant-potential resting process, an LSV scan is not instantaneous but proceeds continuously over a defined potential range. Therefore, during LSV measurements in a CO/H_2_ atmosphere, the scan rate directly determines the time spent by the electrode in the CO-containing environment and within different potential regions, thereby influencing the extent of CO adsorption on Pt and the subsequent HOR polarization response. This effect is expected to be more pronounced at low Pt loadings, where the total number of available active sites is limited and CO-induced site blockage can be more directly reflected in the limiting-current response. Accordingly, a Pt loading of 5 μg_Pt_ cm^−2^ was selected to evaluate the influence of scan rate.

**Figure 4:**
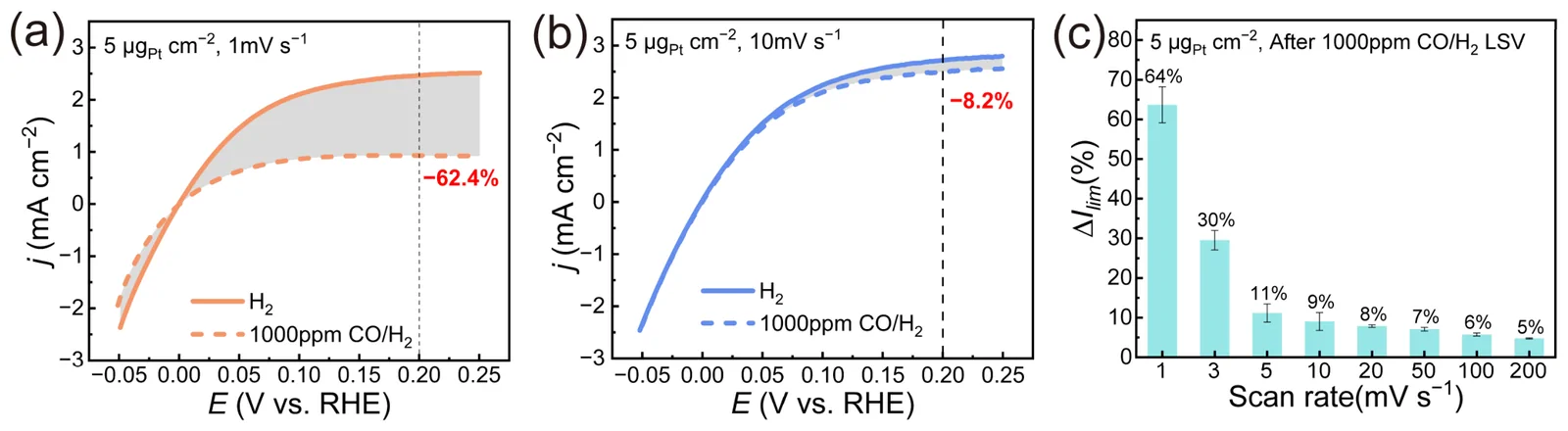
Effect of scan rate on CO tolerance evaluation. (**a**) Comparison of LSV polarization curves recorded at 1 mV s^−1^ in H_2_ and 1000 ppm CO/H_2_ at a Pt loading of 5 μg_Pt_ cm^−2^; (**b**) comparison of LSV polarization curves recorded at 10 mV s^−1^ in H_2_ and 1000 ppm CO/H_2_ at a Pt loading of 5 μg_Pt_ cm^−2^; (**c**) comparison of LSV limiting currents (at 200 mV) measured in H_2_ and 1000 ppm CO/H_2_ at different scan rates. Data points represent the mean of at least five independent trials conducted under identical conditions; error bars denote SDs.

At a Pt loading of 5 μg_Pt_ cm^−2^, HOR polarization curves were recorded at different LSV scan rates in pure H_2_ and in 1000 ppm CO/H_2_ without a prior constant-potential holding step. As shown in Figure 4a, at a scan rate of 1 mV s^−1^, the LSV limiting current of Pt/C in 1000 ppm CO/H_2_ decreased by 62.4% relative to that in pure H_2_. This result indicates that, under slow-scan conditions, the electrode experiences a longer effective exposure time in the CO-containing atmosphere and particularly within the low-overpotential region, allowing CO to adsorb continuously on Pt and progressively occupy HOR active sites, thereby causing a pronounced loss of reaction current. In contrast, when the scan rate was increased to 10 mV s^−1^, the limiting current in the CO-containing atmosphere decreased by only 8.2% (Figure 4b). The faster potential sweep therefore substantially shortened the effective interaction time between CO and the Pt surface, preventing CO poisoning from fully developing and consequently reducing the activity loss observed during the LSV measurement.

The limiting-current decay was further compared over a scan-rate range of 1–200 mV s^−1^. As shown in Figure 4c and Figures S8 and S9, increasing the scan rate from 1 to 5 mV s^−1^ rapidly reduced the limiting-current loss in 1000 ppm CO/H_2_ from 62.4% to approximately 14%. With further increases in scan rate, the current loss remained at a relatively low level of approximately 5–8%, corresponding to an approximately order-of-magnitude reduction in the apparent poisoning-induced current decay compared with that observed at 1 mV s^−1^. This trend demonstrates that LSV scan rate has a pronounced effect on apparent CO tolerance evaluation, particularly in the low-scan-rate region, where relatively small changes in scan rate can lead to substantial differences in limiting-current retention. Similar scan-rate-dependent behavior was also observed at a higher Pt loading of 20 μg_Pt_ cm^−2^, although the effect was less pronounced because the presence of excess active sites partially diluted the impact of CO poisoning (Figures S10 and S11).

Therefore, LSV scan rate should be regarded as a critical parameter in CO tolerance evaluation. A low scan rate prolongs the effective exposure of the electrode to CO/H_2_ and more fully reflects the inhibitory effect of CO adsorption on HOR activity, whereas a high scan rate may underestimate the severity of CO poisoning and thus lead to an apparently higher CO tolerance. Consequently, CO tolerance results obtained using different LSV scan rates should not be directly compared across studies [49,50].

### 3.4. Effect of Catalyst Loading on Apparent CO Tolerance and Interfacial Kinetics

After establishing that both resting time and LSV scan rate influence the apparent CO tolerance response, we further investigated the effect of catalyst loading. Catalyst loading directly determines the total number of Pt active sites available for the HOR. Therefore, under a CO-containing atmosphere, a higher Pt loading may weaken the impact of CO coverage on the macroscopic current response by providing a larger number of remaining available active sites, thereby leading to apparently improved CO tolerance. Previous studies have shown that Pt loading and electrode configuration can significantly affect HOR responses and performance evaluation under CO-containing conditions [51,52,53].

**Figure 5:**
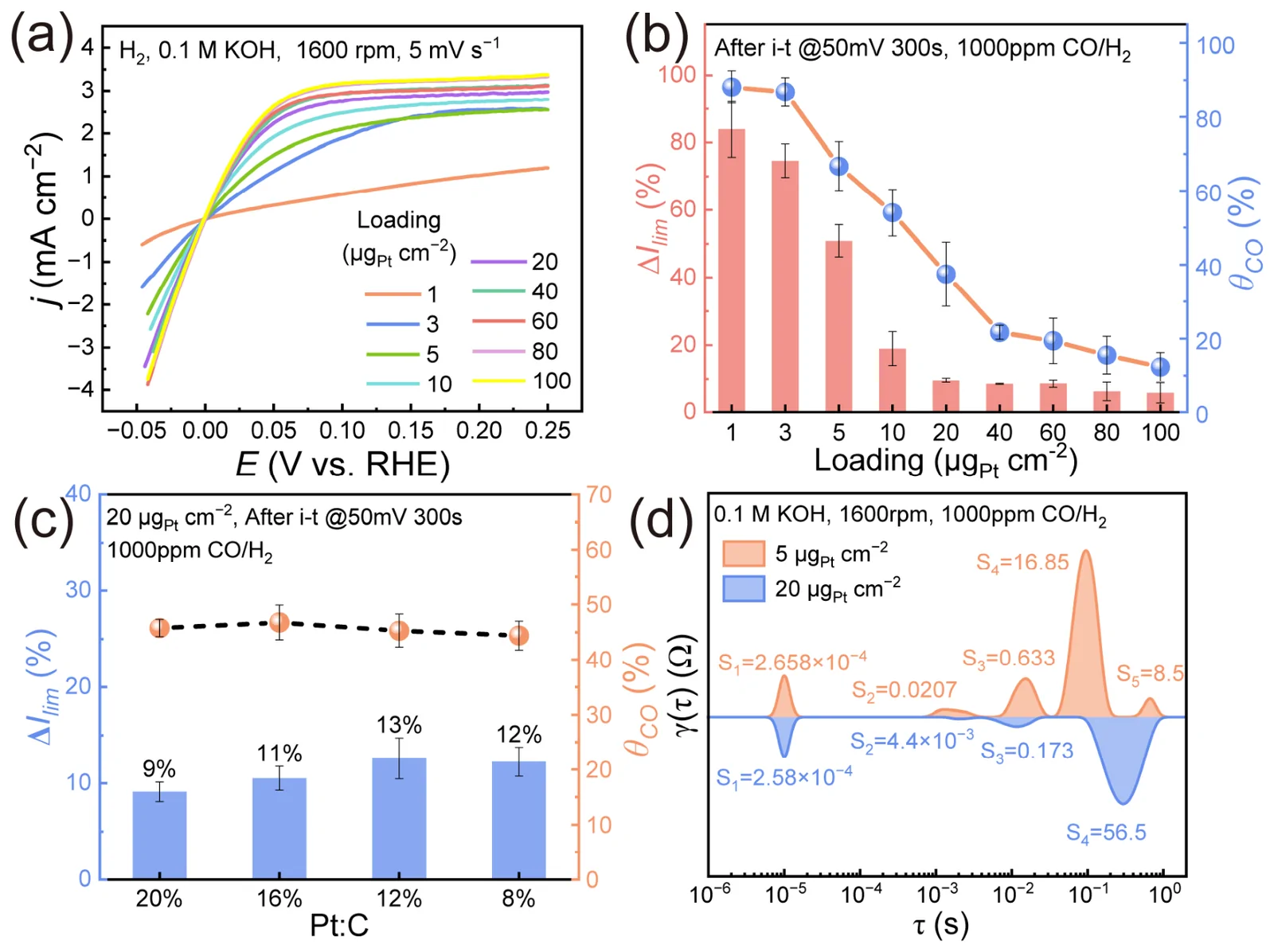
Effect of catalyst loading on CO tolerance evaluation. (**a**) LSV polarization curves at different Pt loadings; (**b**) decrease in LSV limiting current and corresponding CO coverage for electrodes with different Pt loadings after a 300 s hold at 50 mV vs. RHE in 1000 ppm CO/H_2_; (**c**) decrease in LSV limiting current and corresponding CO coverage for catalysts with different Pt mass fractions at the same Pt loading after a 300 s hold at 50 mV vs. RHE in 1000 ppm CO/H_2_; (**d**) distribution of relaxation times (DRT) for electrodes with Pt loadings of 5 and 20 μg_Pt_ cm^−2^ measured at 50 mV vs. RHE in 1000 ppm CO/H_2_. Data points represent the mean of at least five independent trials conducted under identical conditions; error bars denote SDs.

We first compared the LSV polarization curves (Figure 5a) and CV curves (Figure S12) of Pt/C electrodes with different Pt loadings. As the Pt loading increased, the HOR limiting current gradually increased and approached a plateau. This behavior indicates that, in the low-loading regime, the number of Pt active sites is an important factor governing HOR current output. At higher loadings, however, the reaction becomes increasingly limited by factors such as mass transport or electrode utilization efficiency, and further increases in Pt loading provide progressively smaller gains in limiting current. These results demonstrate that electrodes with different Pt loadings possess different numbers of effectively accessible active sites and different current-response capacities and therefore may exhibit substantially different activity-retention values under CO-containing conditions.

The CO tolerance of electrodes with different Pt loadings was then evaluated using an identical testing protocol by comparing the decrease in LSV limiting current in 1000 ppm CO/H_2_ relative to pure H_2_, together with the corresponding post-test CO coverage (Figure 5b and Figures S13–S16). At Pt loadings below 5 μg_Pt_ cm^−2^, the limiting current decreased by more than 50% under the CO-containing atmosphere, while the CO coverage exceeded 70%. This result indicates that, under low-loading conditions, extensive occupation of the limited number of Pt active sites by CO leaves insufficient available sites to sustain the HOR effectively. As the Pt loading increased, the limiting-current decay decreased rapidly and stabilized at approximately 10%, while the CO coverage also showed a decreasing trend. These results suggest that higher-loading electrodes contain a larger number of Pt sites available for reaction. Even when part of the surface is occupied by CO, the remaining active sites can still sustain a substantial fraction of the HOR current, thereby reducing the apparent poisoning effect observed in the LSV response [52,53]. Constant-potential stability tests at different Pt loadings exhibited a similar trend (Figure S17), further supporting the conclusion that high catalyst loading can dilute or mask the activity loss caused by CO poisoning.

To distinguish the effect of absolute noble-metal loading from that of the total catalyst mass and associated catalyst-layer structure, Pt/C catalysts with different Pt mass fractions were compared at the same absolute Pt loading. At a fixed Pt loading of 20 μg_Pt_ cm^−2^, decreasing the Pt content from 20 to 8 wt% increases the deposited catalyst mass from approximately 100 to 250 μg_cat_ cm^−2^, which is expected to increase the catalyst-layer thickness and modify the associated transport environment while maintaining the same total amount of Pt. As shown in Figure 5c and Figures S18 and S19, the 20%, 16%, 12%, and 8% Pt/C catalysts nevertheless exhibited similar LSV limiting-current losses, mainly within 10–15%, and CO coverages of approximately 40–50%. These results indicate that, under the present RDE conditions, the absolute Pt loading and the resulting number of available Pt sites are the dominant factors governing the apparent CO tolerance response, whereas changes in catalyst-layer thickness and transport make comparatively smaller contributions.

The influence of CO poisoning on interfacial kinetic processes at different Pt loadings was further analyzed by EIS combined with DRT analysis [54,55]. As shown in Figure 5d, electrodes with Pt loadings of 5 and 20 μg_Pt_ cm^−2^ exhibited markedly different DRT response features in 1000 ppm CO/H_2_. Compared with the low-loading electrode, the high-loading electrode showed smaller impedance contributions associated with charge transfer and intermediate adsorption/desorption processes, indicating that increasing the Pt loading provides a larger number of available reaction sites and thereby alleviates the inhibition of HOR interfacial processes in the presence of CO. Meanwhile, the impedance contribution associated with mass transport increased for the high-loading electrode and shifted toward longer relaxation times. This behavior may be related to changes in gas diffusion, electrolyte penetration, and reactant transport pathways within the thicker catalyst layer [55,56,57]. The corresponding Nyquist and Bode plots, together with the equivalent-circuit fitting results, are provided in Figures S20–S22 and Table S2. The impedance spectra were fitted using an equivalent circuit of R_s_-CPE1||(R_1_-(CPE2||R_2_)). In this model, CPE1 represents the non-ideal double-layer capacitance associated with the heterogeneous Pt/electrolyte interface, while R_1_ is related to the charge-transfer process of HOR. The CPE2||R_2_ element describes a slower adsorbate-related interfacial relaxation process during HOR. At 5 μg_Pt_ cm^−2^, CO exposure markedly increases the impedance contributions associated with interfacial charge transfer and intermediate adsorption/desorption. In contrast, the additional impedance induced by CO progressively decreases with increasing Pt loading, consistent with the availability of excess Pt sites that can sustain the HOR despite partial CO occupation.

Overall, catalyst loading is a critical parameter governing the CO tolerance evaluation of HOR catalysts. Low-loading electrodes are more sensitive to CO coverage, because even limited CO adsorption can substantially reduce the number of available active sites and cause pronounced current loss. By contrast, high-loading electrodes contain more remaining available Pt sites and may therefore exhibit a weaker poisoning effect in the macroscopic LSV response. Importantly, this reduced apparent poisoning at high loading should not be interpreted as an improvement in the catalyst-intrinsic resistance to CO poisoning, because the physicochemical interaction between CO and the catalyst surface has not necessarily been altered; instead, the reduced current loss may arise from the masking of CO poisoning by an excess of available active sites [52,53]. Therefore, CO tolerance results obtained at different noble-metal loadings may not be directly comparable across studies. For standardized CO tolerance evaluation, catalyst loading must be strictly controlled and clearly reported; otherwise, loading-induced apparent activity retention may be mistakenly interpreted as an enhancement in catalyst-intrinsic resistance to CO poisoning.

### 3.5. Recommended Protocol for Evaluating the CO Tolerance of HOR Catalysts

Based on the systematic investigations described above, we find that the apparent CO tolerance of HOR catalysts is strongly dependent on the testing conditions. The constant-potential resting time before LSV determines the extent of CO pre-adsorption, the LSV scan rate controls the effective exposure time during the dynamic measurement, and catalyst loading determines whether the apparent poisoning effect is amplified or diluted by altering the number of remaining available active sites. Therefore, establishing a standardized evaluation protocol is essential for ensuring meaningful comparison of CO tolerance performance among different catalysts [50,58,59].

**Figure 6:**
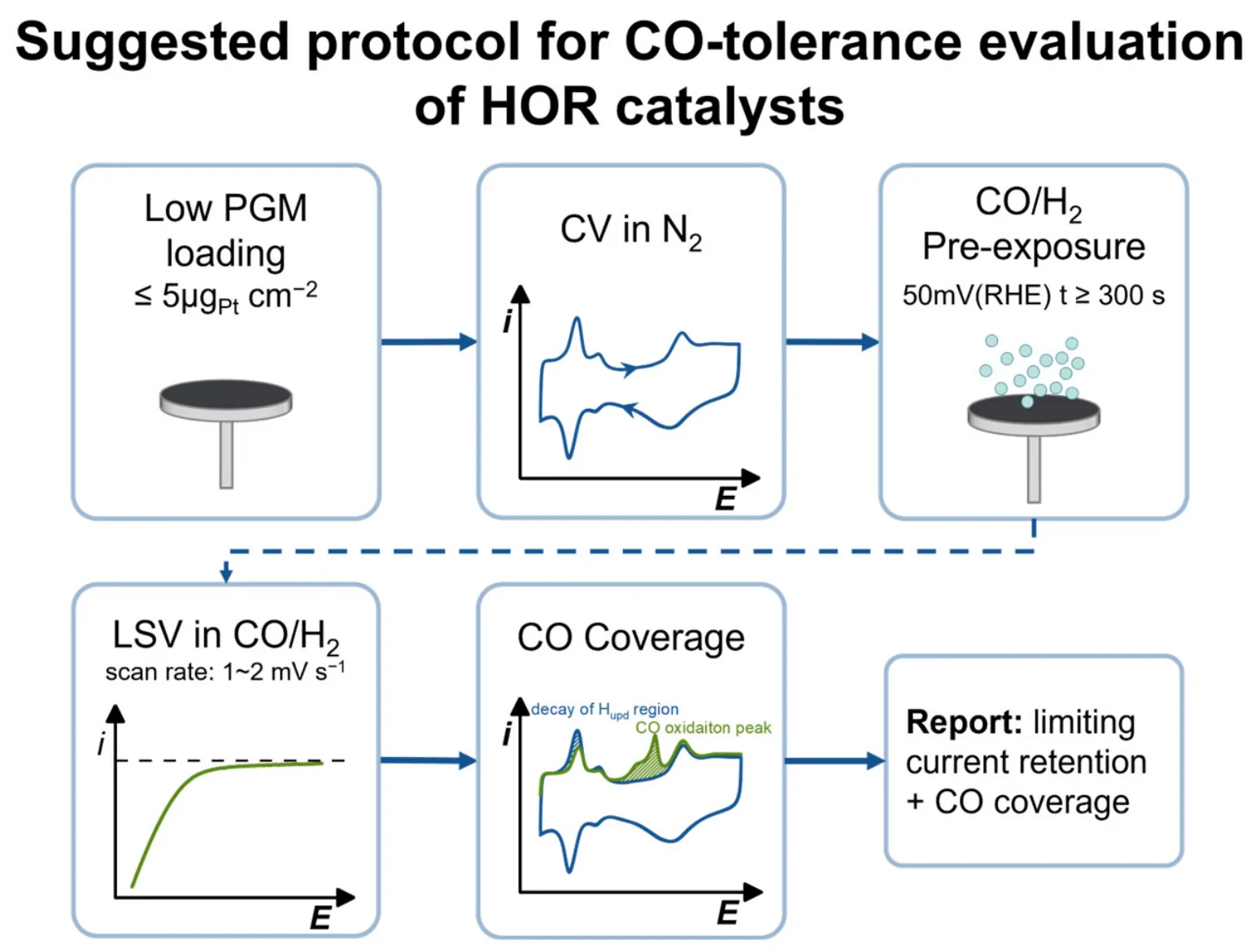
Recommended protocol for CO tolerance evaluation of HOR catalysts.

For CO tolerance evaluation based primarily on LSV measurements, the following protocol is recommended (Figure 6). For other PGM-based HOR catalysts, rather than directly adopting the same numerical loading threshold, a sufficiently low loading regime should first be experimentally identified to avoid masking of CO poisoning by excess available sites. Second, after electrochemical activation and before the LSV measurement in CO/H_2_, a clearly defined constant-potential pre-exposure step should be introduced. We recommend holding the electrode within the HOR potential region, for example at 50–100 mV vs. RHE, for no less than 300 s to allow CO adsorption to approach a quasi-steady state. A holding potential of 5 mV vs. RHE may be adopted as a standardized condition. Although our results show that potential variations within the 0–200 mV overpotential range exert only a limited influence on CO coverage and limiting-current decay, the use of a fixed potential can eliminate potential protocol-dependent bias. Third, a low and standardized LSV scan rate, preferably 1–2 mV s^−1^, should be employed to prolong the effective exposure time of the electrode to the CO-containing atmosphere and better capture the inhibitory effect of CO adsorption on HOR activity. Excessively high scan rates substantially shorten the effective interaction time between CO and the Pt surface, preventing the poisoning process from fully developing and thereby underestimating the actual sensitivity of the catalyst to CO poisoning. Fourth, post-test CV analysis should be performed to estimate the effective CO coverage from the suppression of the H_upd_ desorption charge before and after CO exposure. This site-blocking metric directly reflects the fraction of Pt sites rendered unavailable for hydrogen adsorption and enables the relationship between apparent current loss and CO-induced active-site occupation to be evaluated quantitatively.

For stability evaluation based on constant-potential chronoamperometry, the noble-metal loading should likewise be strictly controlled, preferably at no more than 5 μg_Pt_ cm^−2^. A clearly defined holding potential, such as 50 mV vs. RHE, should be used together with a standardized CO concentration and testing duration. Reported studies have employed CO concentrations ranging from 100 to 10,000 ppm and testing durations ranging from several hundred seconds to several hours. In the present work, we recommend 1000 ppm CO as a standardized testing concentration and a measurement duration of no less than 1000 s to sufficiently evaluate activity decay under continuous CO exposure. The i–t measurement should be accompanied by a corresponding control experiment in pure H_2_ so that activity loss caused by CO poisoning can be distinguished from intrinsic catalyst degradation during the test.

It should be emphasized that the recommendations above are not intended to prescribe a single mandatory combination of testing parameters for all HOR catalysts. The specific numerical conditions proposed here were established using Pt/C as a model catalyst under the present alkaline RDE configuration. Catalysts with substantially different CO adsorption/desorption kinetics, electrochemically active surface areas, bifunctional CO-removal pathways, metal–support interactions, or catalyst-layer structures may require adjustment of the catalyst loading and CO pre-exposure duration to achieve an appropriate and measurable poisoning state. Therefore, for other Pt- or PGM-based catalysts, the general principle of employing a sufficiently sensitive catalyst loading, adequate CO pre-exposure, and a controlled slow potential scan is expected to remain applicable, whereas the exact numerical thresholds should be validated for the catalyst system under investigation.

Although such a protocol requires several experimental variables to be controlled, this additional constraint should be viewed in the context of the much larger variability in currently reported CO tolerance measurements. As summarized in Table 1, catalyst loading, CO concentration, scan rate, and exposure conditions vary substantially among published studies, making direct comparison of the reported activity-retention values difficult. A predefined and consistently reported set of testing parameters therefore provides a more reproducible and cross-study-comparable framework than the current evaluation practices. Accordingly, the protocol proposed here should be regarded as a standardized RDE screening framework rather than a universal fixed recipe. It is intended to minimize protocol-induced bias during preliminary catalyst comparison, while subsequent validation under MEA or practical fuel-cell operating conditions remains necessary for assessing device-level CO tolerance.

## 4. Conclusions

In summary, this work employed structurally well-defined Pt/C with a uniform particle size as a model catalyst to systematically investigate the influence of RDE testing parameters on the apparent CO tolerance evaluation of alkaline HOR catalysts. First, LSV and constant-potential stability tests produced markedly different poisoning responses for the same Pt/C catalyst in 1000 ppm CO/H_2_, demonstrating that experimentally measured activity retention is a protocol-dependent apparent response and should not be directly equated with the catalyst-intrinsic resistance to CO poisoning.

Further investigation revealed that the constant-potential resting period before LSV scanning effectively functions as a CO pre-exposure stage. Prolonging this resting time increased CO coverage on the Pt surface and progressively suppressed the subsequent LSV limiting current, whereas variation of the holding potential within the examined low-overpotential range exerted a comparatively minor influence. The LSV scan rate was identified as another decisive parameter: slower scanning prolonged the effective exposure of the electrode to the CO/H_2_ atmosphere, thereby amplifying the apparent poisoning effect, while overly fast scanning risked underestimating the extent of CO poisoning.

Catalyst loading also emerged as a critical factor governing apparent CO tolerance. At low Pt loadings, CO adsorption substantially depleted the available active sites and caused severe current decay. Conversely, high Pt loadings could dilute or mask the effect of CO poisoning by providing excess active sites, thereby yielding artificially enhanced apparent CO tolerance. EIS/DRT analysis further revealed distinct, loading-dependent effects of CO on charge-transfer, intermediate adsorption/desorption, and mass-transport processes, providing mechanistic insight into how loading governs the poisoning response.

Based on these findings, we recommend that standardized CO tolerance evaluation of HOR catalysts should, as far as possible, adopt consistent noble-metal loading, CO concentration, pre-scan constant-potential holding time, holding potential, and LSV scan rate, combined with post-test analysis of CO coverage. This work demonstrates that a non-negligible portion of the variation in reported CO tolerance performance may originate from differences in testing protocols in addition to genuine differences in catalyst-intrinsic physicochemical properties. Our results thus provide an experimental basis for developing more reliable and cross-study-comparable methodologies for evaluating the CO tolerance of HOR catalysts.

## Figures and Tables

**Table 1:** Summary of CO tolerance evaluation protocols reported for HOR catalysts.

Num.	Catalyst	Loading μg_PGM_ cm^−2^	LSV		Pt/C Loss (%)	Ref.
			CO (ppm)	Scan Rate (mV s^−1^)		
This work	Pt/C	5	1000	1	64	-
	Pt/C	5	1000	5	11	-
	Pt/C	20	1000	1	15	-
	Pt/C	20	1000	10	9.1	-
1	PtMo/MoO_x_	9.4	100	5	-	[32]
2	PtW-WO_3_/C	5	1000	5	47	[33]
3	ZnIn-PtRu/C	2	1000	50	30	[34]
4	Mo-Pt/NC	10	1000	10	-	[35]
5	Pt-MoC@NC	10	1000	5	37	[36]
6	Pt_3_Sn	7.9	100	10	44.7	[37]
7	Pt_1_@Co_1_CN	11.2	1000	10	54	[38]
8	Rh_2_Sb/CNT	12	1000	5	73.8	[39]
9	RhSb_2_/CNT	12	1000	5	73.8	[39]
10	Ru@C-280	10	1500/20,000	1	-	[40]
11	Ru@C	10	1500/20,000	1	-	[40]
12	PtRu/C	10	1500/20,000	1	-	[40]
13	Pt/C	10	1500/20,000	1	87/100	[40]
14	Ru-Cr(OH)_x_	60	20,000	2	100	[41]
15	Co_1_Ru_1,n_/rGO	5.5	1000	5	78	[42]
16	Ru@TiO_2_	25	1000	10	-	[43]
17	Ru/Mg_s_/C	20	1000	1	-	[44]
18	s-Ru_1_Pt@W_1_/NC	6.27–12.54	1000	10	47.5	[45]
19	O-Pt_3_In/rGO	2	2000	5	80.7	[46]
20	PtNiMo/Fe-N-C	15	1000	5	66.1	[47]

Note: For the linear sweep voltammetry (LSV) measurements in this work, the reported CO tolerance values at different scan rates were obtained without CO pre-exposure; 1500/20,000 indicates that tests have been conducted for both types of CO content.

## Data Availability

The original contributions presented in this study are included in the article/Supplementary Material. Further inquiries can be directed to the corresponding author.

## Supplementary Materials

The following supporting information can be downloaded at https://www.mdpi.com/article/10.3390/ma19163460/s1, Section S1: Experimental Methods; Section S1.1: Physical characterization; Section S1.2: The dilution effect of CO; Section S2: Supplementary Figures; Figure S1: (a) Transmission electron microscopy (TEM) image of Pt/C; (b) particle size distribution statistics; (c) High-resolution TEM image of Pt/C; Figure S2: X-ray photoelectron spectroscopy (XPS) survey spectra of Pt/C; Figure S3: Cyclic voltammetry (CV) curves for carbon support and Pt/C under N_2_ atmosphere and CO adsorption(a) and linear sweep voltammetry (LSV) curves (b); Figure S4: Specific activity (SA) and mass activity (MA) (a), turnover frequency (TOF) (b) of Pt/C; Figure S5: (a) LSV of Pt/C in H_2_; (b) LSV after CO poisoning; (c) CO coverage after 300 s i−t poisoning and subsequent LSV measurement; Figure S6: Comparison of LSV before and after 300 s CO poisoning at an overpotential of 50 mV (a), 100 mV (b) and 150 mV (c) vs. RHE; Figure S7: Comparison of CV before and after 300 s CO poisoning at an overpotential of 0 mV (a), 50 mV (b), 100 mV (c), 150 mV (d), and 200 mV (e) vs. RHE; Figure S8: LSV recorded at various scan rates under H_2_ (a) and 1000 ppm CO/H_2_ (b) with a Pt loading of 5 μg_Pt_ cm^−2^; Figure S9: Comparison of LSV measured under H_2_ and 1000 ppm CO/H_2_ at scan rates of 3 mV s^−1^ (a), 5 mV s^−1^ (b), 20 mV s^−1^ (c), 50 mV s^−1^ (d), 100 mV s^−1^ (e), and 200 mV s^−1^ (f) with a catalyst loading of 5 μg_Pt_ cm^−2^; Figure S10: Comparison of LSV measured under H_2_ and 1000 ppm CO/H_2_ at scan rates of 1 mV s^−1^ (a), 3 mV s^−1^ (b), 5 mV s^−1^ (c), 10 mV s^−1^ (d), 20 mV s^−1^ (e), and 50 mV s^−1^ (f) with a catalyst loading of 20 μg_Pt_ cm^−2^; Figure S11: Comparison of LSV measured under H_2_ and 1000 ppm CO/H_2_ at scan rates of 100 mV s^−1^ (a) and 200 mV s^−1^ (b) with a catalyst loading of 20 μg_Pt_ cm^−2^; LSV at various scan rate under pure H_2_ (c) and 1000 ppm CO/H_2_ (d); (e) Comparison of LSV limiting currents obtained in H_2_ and 1000 ppm CO/H_2_ at different scan rates; Figure S12: CV curves with different catalyst loadings; Figure S13: CO poisoning for 300 s at an overpotential of 50 mV vs. RHE for catalysts with different loadings under 1000 ppm CO/H_2_; Figure S14: Comparison of CV curves (a–c) and LSV curves (d–f) before and after 300 s i−t testing at an overpotential of 50 mV vs. RHE under 1000 ppm CO/H_2_ with catalyst loading of 1, 3 and 5 μg_Pt_ cm^−2^, respectively. Figure S15: Comparison of CV curves (a–c) and LSV curves (d–f) before and after 300 s i−t testing at an overpotential 50 mV vs. RHE under 1000 ppm CO/H_2_ with catalyst loading of 10, 20 and 40 μg_Pt_ cm^−2^, respectively. Figure S16: Comparison of CV curves (a–c) and LSV curves (d–f) before and after 300 s i−t testing at an overpotential 50 mV vs. RHE under 1000 ppm CO/H_2_ with catalyst loading of 60, 80 and 100 μg_Pt_ cm^−2^, respectively; Figure S17: Stability tests for 1000 s at an overpotential of 50 mV vs. RHE under 1000 ppm CO/H_2_ for catalyst with different loadings; Figure S18: Comparison of LSV before and after 300 s i−t tests at an overpotential of 50 mV vs. RHE under 1000 ppm CO/H_2_ for Pt/C catalysts with Pt mass fractions of 8% (a), 12% (b), 16% (c) and 20% (d) at a noble metal loading of 20 μg_Pt_ cm^−2^; Figure S19. Comparison of CV before and after 300 s i−t measurements at an overpotential of 50 mV vs. RHE under 1000 ppm CO/H_2_ for Pt/C catalysts with Pt mass fractions of 8% (a), 12% (b), 16% (c) and 20% (d) at a noble metal loading of 20 μg_Pt_ cm^−2^; Figure S20: Nyquist plots (a) and corresponding Bode plots (b) of Pt/C recorded at different applied potentials; (c) The fitted equivalent circuit diagram; Figure S21. Comparison of Nyquist plots (a–c) and Bode plots (d–f) of Pt/C with catalyst loadings of 5, 10, and 20 μg_Pt_ cm^−2^ at an overpotential of 50 mV in pure H_2_ and 1000 ppm CO/H_2_ atmosphere; Figure S22. Comparison of Nyquist plots (a–c) and Bode plots (d–f) of Pt/C with catalyst loadings of 40, 60, and 80 μg_Pt_ cm^−2^ at an overpotential of 50 mV in pure H_2_ and 1000 ppm CO/H_2_ atmosphere; Section S3. Supplementary Tables; Table S1: ICP results for the prepared samples; Table S2: EIS fitting results.
