# Supplementary material for: Parameter Decoupling and Standardization of an RDE Protocol for CO Tolerance Evaluation of the Alkaline Hydrogen Oxidation Reaction
Source: Materials (Basel). 2026 Aug 14;19(16):3460. doi: 10.3390/ma19163460 (PMC13514353; doi:10.3390/ma19163460)
Supplement: Supplementary file 1 [file materials-19-03460-s001.zip › materials-4453800-supplementary.pdf]

Supplementary Materials

Parameter Decoupling and Standardization of an RDE Protocol for CO Tolerance Evaluation of Alkaline Hydrogen Oxidation Reaction

Hong-Fei Xing[1], Mei-Li Wang[1], Hao-Ran Wu[1], Wei-Dong Li[1] and Bang-An Lu[1,*]

[1] College of Materials Science and Engineering, Zhengzhou University, Zhengzhou 450001, P. R. China.

* Correspondence: balu@zzu.edu.cn

S1. Experimental Methods

S1.1. Physical characterization

Transmission electron microscopy (TEM), high-angle annular dark-field scanning transmission electron microscopy (HAADF-STEM), and energy-dispersive X-ray spectroscopy (EDS) elemental mapping were performed on an FEI Talos F200X microscope operated at 200 kV(USA).

Powder X-ray diffraction (XRD) patterns were collected on an X'Pert PRO MPD diffractometer(USA). X-ray photoelectron spectroscopy (XPS) measurements were performed using a Thermo ESCALAB 250Xi spectrometer(USA) equipped with an Al Kα radiation source.

The Pt and Mn contents were quantified by inductively coupled plasma optical emission spectrometry (ICP-OES) using an Agilent 5110 instrument(USA).

Synchrotron X-ray absorption fine structure (XAFS) measurements were performed at the 1W1B beamline of the Beijing Synchrotron Radiation Facility (BSRF). The storage ring was operated at 2.5 GeV with an average current of 250 mA. A Si(111) double-crystal monochromator was employed for energy selection. The X-ray absorption near-edge structure (XANES) and extended X-ray absorption fine structure (EXAFS) spectra at the Pt L_3-edge (11.564 keV) were collected in transmission/fluorescence mode using ionization chambers. All spectra were collected at ambient conditions. Energy calibration was performed using a Pt foil for the Pt L_3-edge. The acquired EXAFS data was processed according to standard procedures using the ATHENA module implemented in the IFEFFIT software packages. The k_3-weighted EXAFS spectra were obtained by subtracting the post-edge background from the overall absorption and then normalizing the edge-jump step.

S1.2. The dilution effect of CO.

Henry's law describes the proportional relationship between the solubility C of a gas in liquid and its partial pressure P:

$$C = K_H P \tag{S1}$$

For a gas mixture containing 1000 ppm CO, the partial pressure of H_2 is 0.999 atm, such that

$$C_{H_2} = K_{H,H_2} \times 0.999\ atm \tag{S2}$$

The Henry's law constants are $K_{H,H_2} = 7.8 \times 10^{-3}\ mol\ L^{-1} atm^{-1}$ for hydrogen and $K_{H,CO} = 9.5 \times 10^{-3} mol\ L^{-1} atm^{-1}$ for carbon monoxide.

According to the Koutecky-Levich(K-L) equation:

$$I_{lim} = 0.62 nFAD^{2/3} v^{-1/6} \omega^{1/2} C \tag{S3}$$

The limiting current I_{lim} is linearly proportional to the gas solubility C. The ratio of limiting currents can be expressed as:

$$\frac{I_{lim,CO/H_2}}{I_{lim,H_2}} = \frac{C_{H_2,CO/H_2}}{C_{H_2,pure}} = \frac{P_{H_2(1000\ ppm\ CO)}}{P_{H_2(pure)}} \tag{S4}$$

Accordingly, the actual limiting current measured under the 1000 ppm CO atmosphere follows:

$$I_{lim} = \frac{I_{lim,CO/H_2}}{0.999} \tag{S5}$$

This result demonstrates that the dilution effect introduced by 1000 ppm CO is negligible.

S2. Supplementary Figures

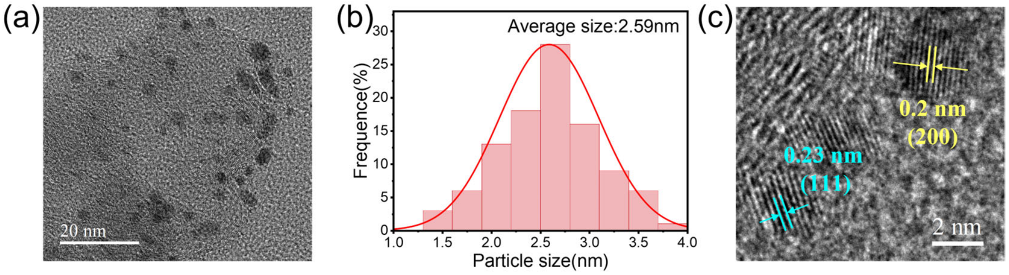

Figure S1. (a) Transmission electron microscopy(TEM) image of Pt/C; (b) particle size distribution statistics; (c) High-resolution TEM image of Pt/C.

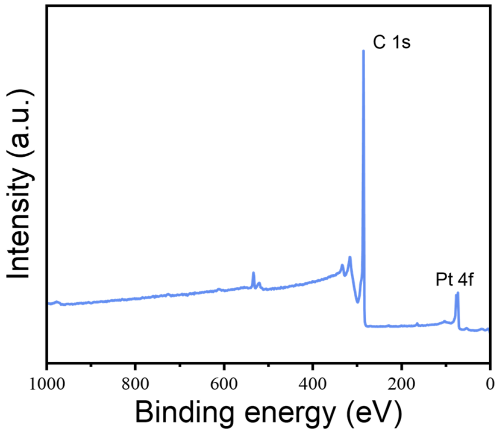

Figure S2. X-ray photoelectron spectroscopy(XPS) survey spectra of Pt/C.

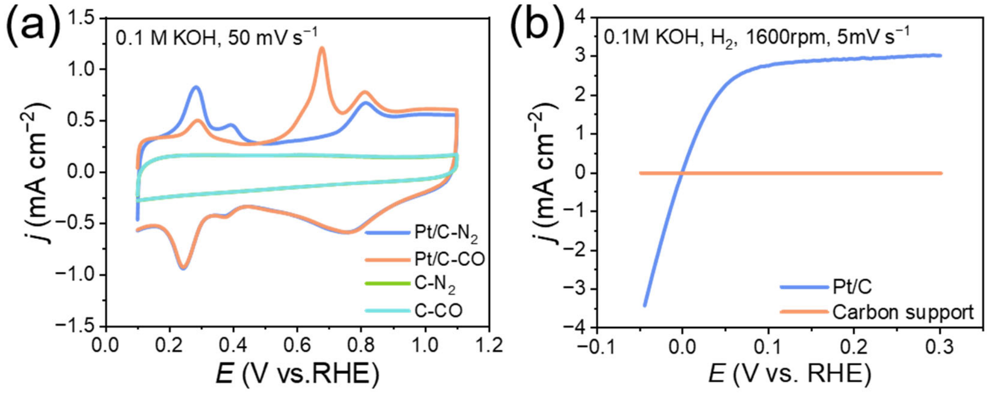

Figure S3. Cyclic voltammetry(CV) curves for carbon support and Pt/C under N_2 atmosphere and CO adsorption(a) and linear sweep voltammetry(LSV) curves(b).

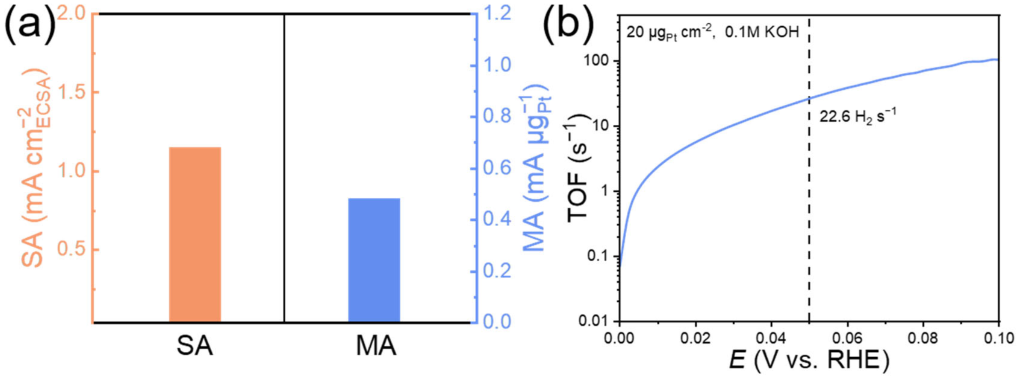

Figure S4. Specific activity(SA) and mass activity(MA)(a), turnover frequency(TOF)(b) of Pt/C.

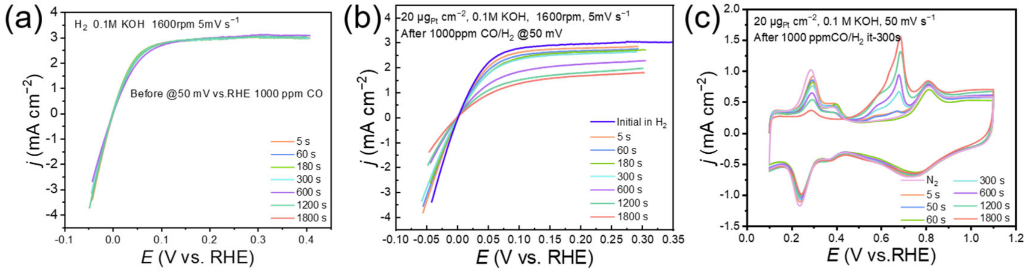

Figure S5. (a) LSV of Pt/C in H_2; (b) LSV after CO poisoning; (c) CO coverage after 300 s i−t poisoning and subsequent LSV measurement.

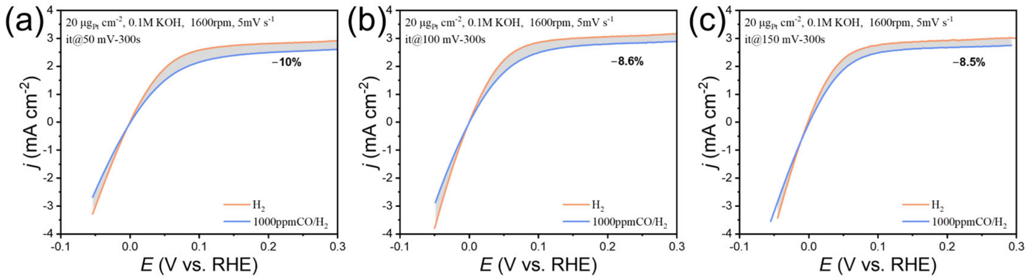

Figure S6. Comparison of LSV before and after 300 s CO poisoning at an overpotential of 50 mV (a), 100 mV (b) and 150 mV (c) vs. RHE.

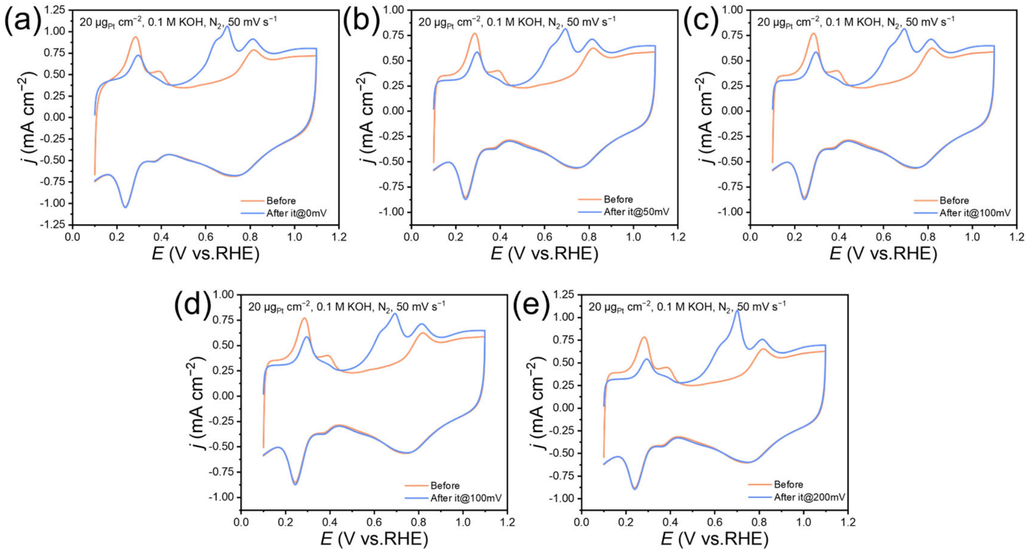

Figure S7. Comparison of CV before and after 300 s CO poisoning at an overpotential of 0 mV (a), 50 mV (b), 100 mV (c), 150 mV (d) and 200 mV (e) vs. RHE.

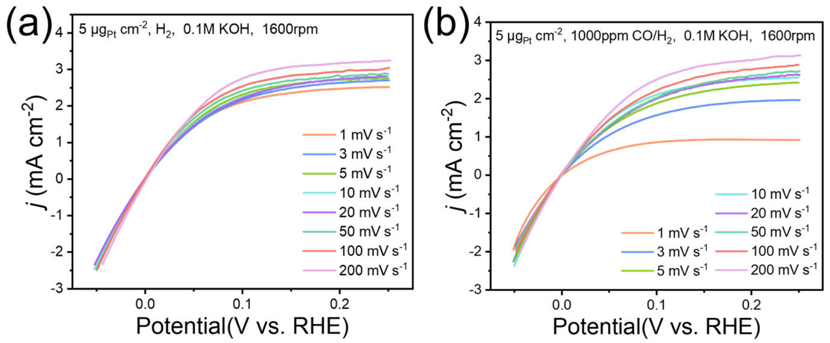

Figure S8. LSV recorded at various scan rates under H_2 (a) and 1000 ppm CO/H_2 (b) with a Pt loading of 5 μg_{Pt} cm^{-2}.

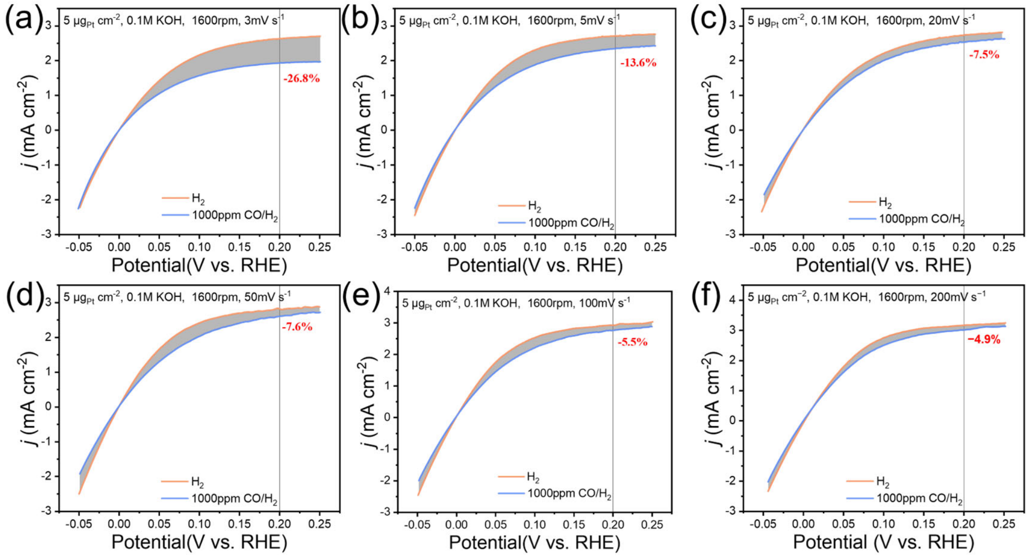

Figure S9. Comparison of LSV measured under H_2 and 1000 ppm CO/H_2 at scan rates of 3 mV s^{-1} (a), 5 mV s^{-1} (b), 20 mV s^{-1} (c), 50 mV s^{-1} (d), 100 mV s^{-1} (e) and 200 mV s^{-1} (f) with a catalyst loading of 5 μg_{Pt} cm^{-2}.

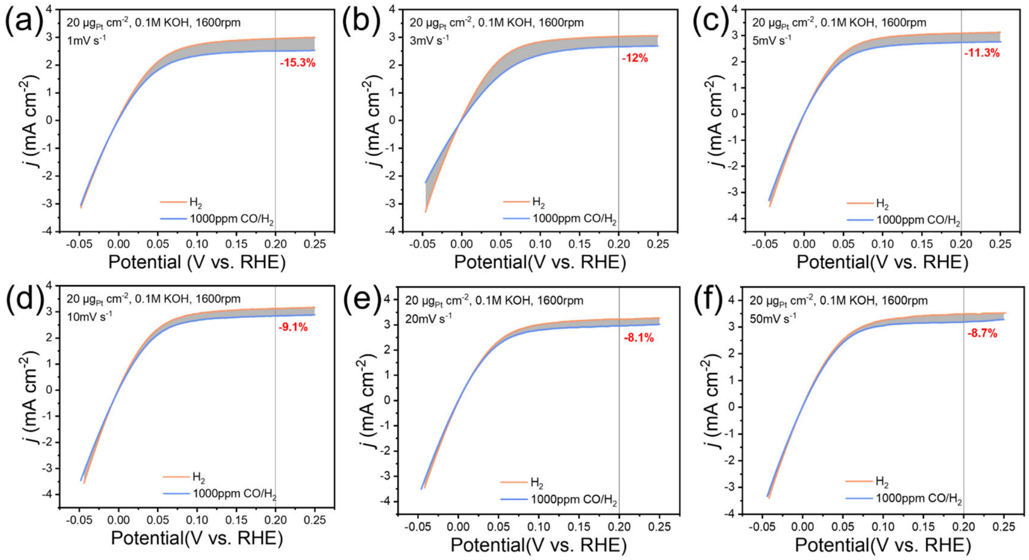

Figure S10. Comparison of LSV measured under H_2 and 1000 ppm CO/H_2 at scan rates of 1 mV s^{-1} (a), 3 mV s^{-1} (b), 5 mV s^{-1} (c), 10 mV s^{-1} (d), 20 mV s^{-1} (e) and 50 mV s^{-1} (f) with a catalyst loading of 20 μg_{Pt} cm^{-2}.

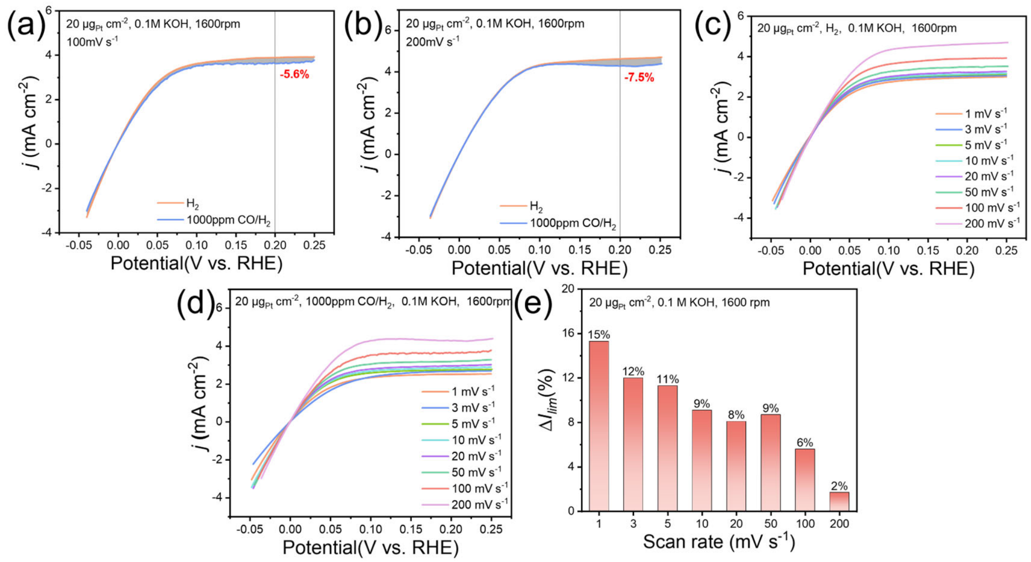

Figure S11. Comparison of LSV measured under H_2 and 1000 ppm CO/H_2 at scan rates of 100 mV s^{-1} (a) and 200 mV s^{-1} (b) with a catalyst loading of 20 μg_{Pt} cm^{-2}; LSV at various scan rate under pure H_2 (c) and 1000 ppm CO/H_2 (d); (e) Comparison of LSV limiting currents obtained in H_2 and 1000 ppm CO/H_2 at different scan rates.

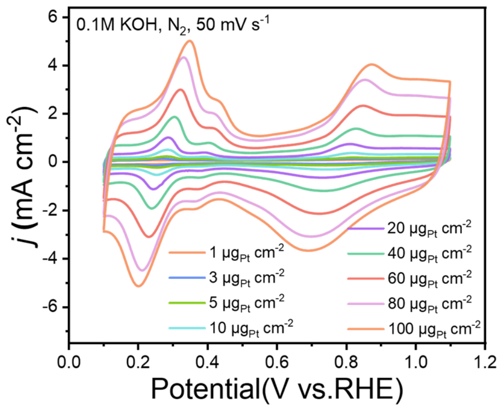

Figure S12. CV curves with different catalyst loadings.

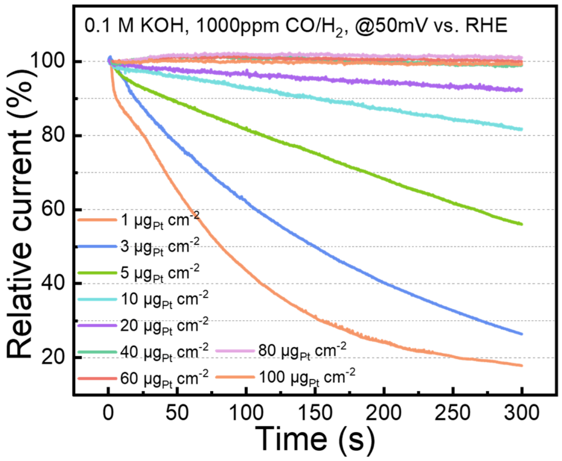

Figure S13. CO poisoning for 300 s at an overpotential of 50 mV vs. RHE for catalysts with different loadings under 1000 ppm CO/H_2.

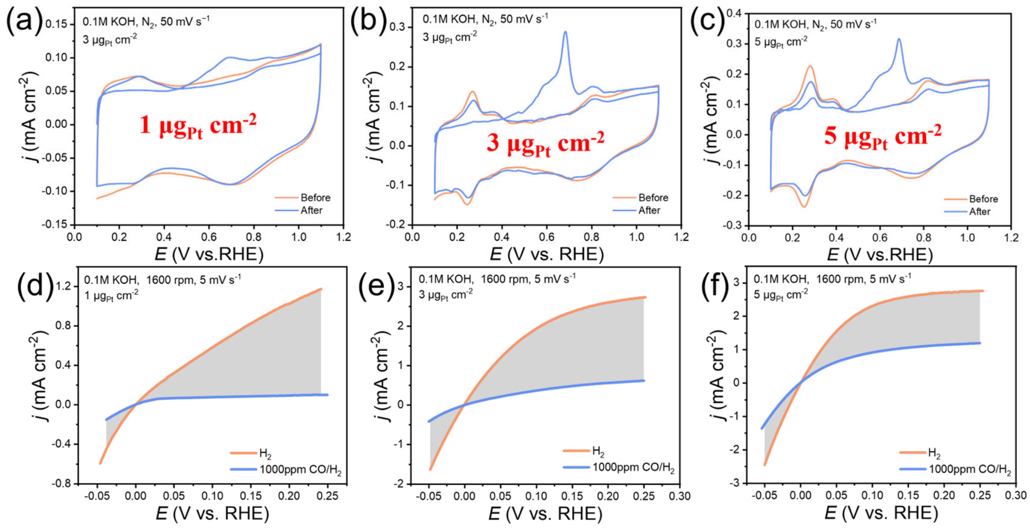

Figure S14. Comparison of CV curves (a-c) and LSV curves (d-f) before and after 300 s i−t testing at an overpotential of 50 mV vs. RHE under 1000 ppm CO/H_2 with catalyst loading of 1, 3 and 5 μg_{Pt} cm^{-2}, respectively.

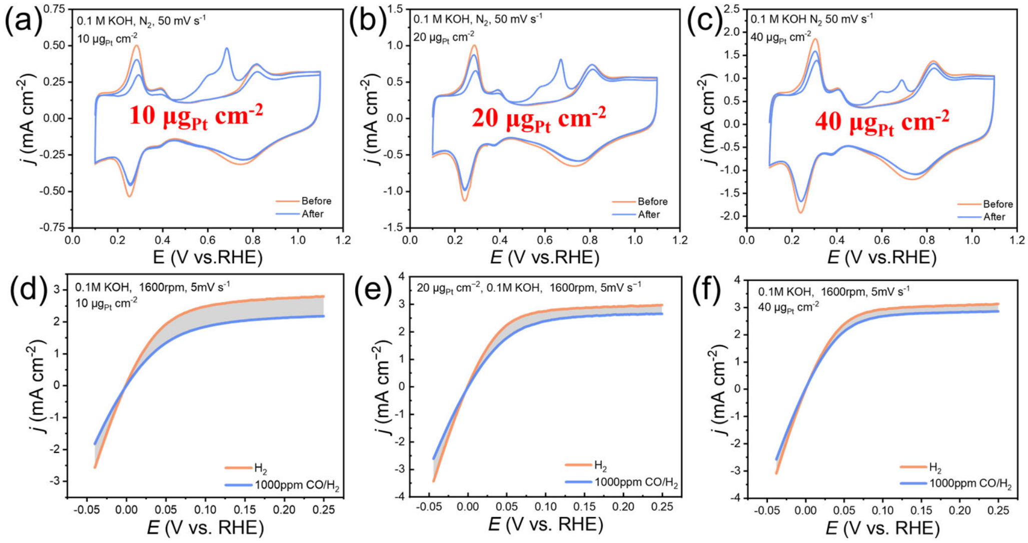

Figure S15. Comparison of CV curves (a-c) and LSV curves (d-f) before and after 300 s i−t testing at an overpotential 50 mV vs. RHE under 1000 ppm CO/H_2 with catalyst loading of 10, 20 and 40 μg_{Pt} cm^{-2}, respectively.

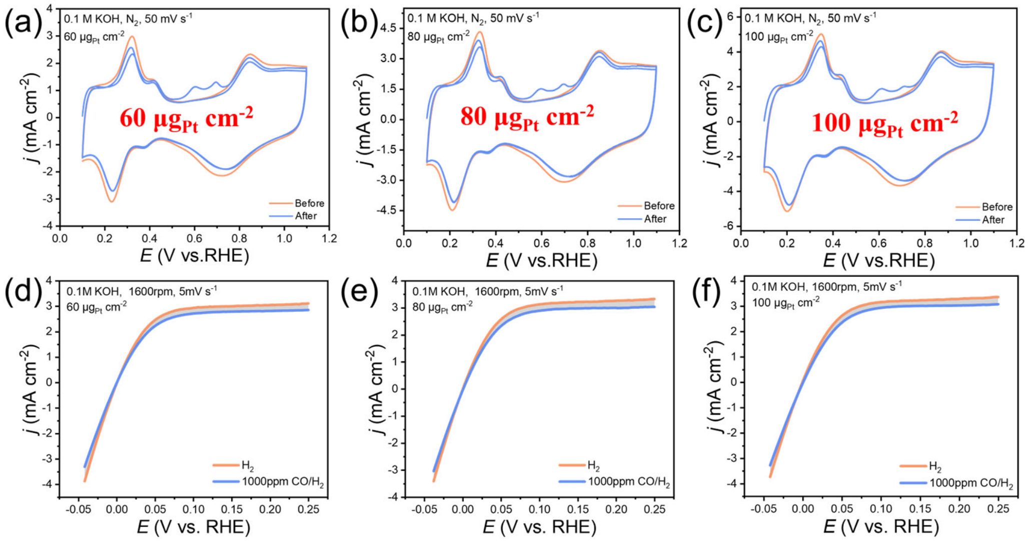

Figure S16. Comparison of CV curves (a-c) and LSV curves (d-f) before and after 300 s i−t testing at an overpotential 50 mV vs. RHE under 1000 ppm CO/H_2 with catalyst loading of 60, 80 and 100 μg_{Pt} cm^{-2}, respectively.

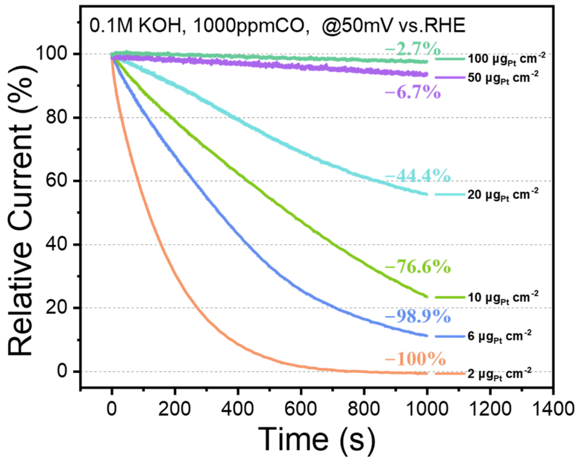

Figure S17. Stability tests for 1000 s at an overpotential of 50 mV vs. RHE under 1000 ppm CO/H_2 for catalyst with different loadings.

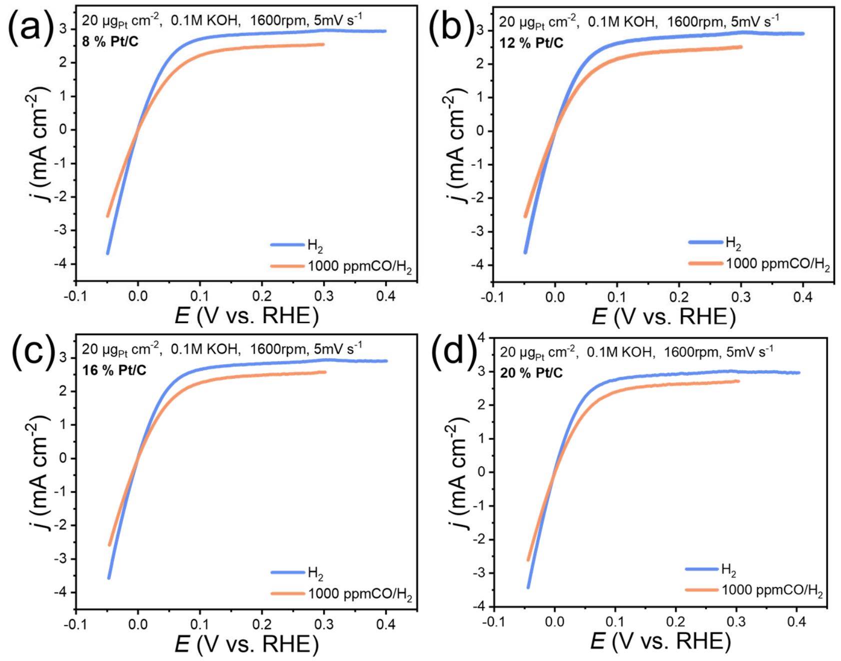

Figure S18. Comparison of LSV before and after 300 s i−t tests at an overpotential of 50 mV vs. RHE under 1000 ppm CO/H_2 for Pt/C catalysts with Pt mass fractions of 8% (a), 12% (b), 16% (c) and 20% (d) at a noble metal loading of 20 μg_{Pt} cm^{-2}.

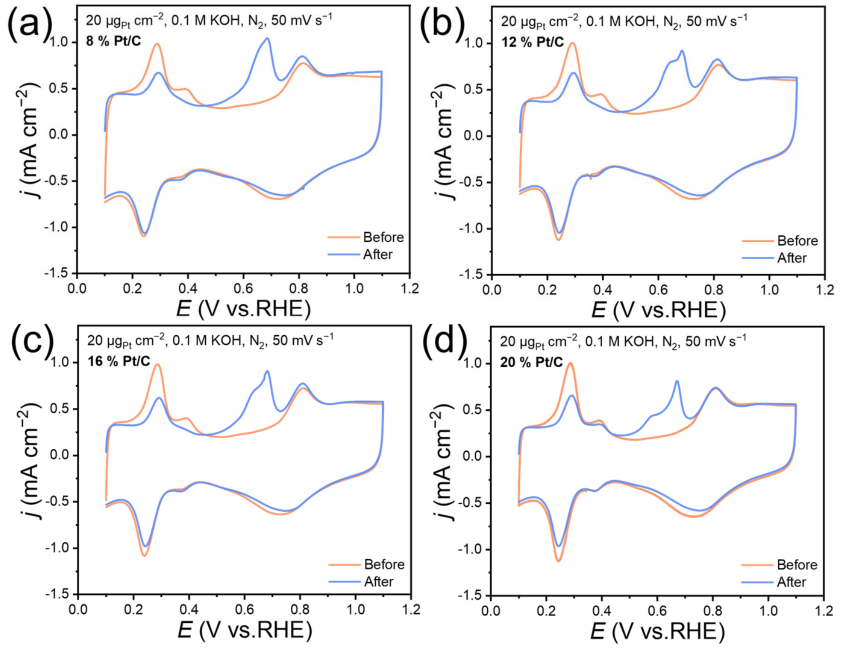

Figure S19. Comparison of CV before and after 300 s i−t measurements at an overpotential of 50 mV vs. RHE under 1000 ppm CO/H_2 for Pt/C catalysts with Pt mass fractions of 8% (a), 12% (b), 16% (c) and 20% (d) at a noble metal loading of 20 μg_{Pt} cm^{-2}.

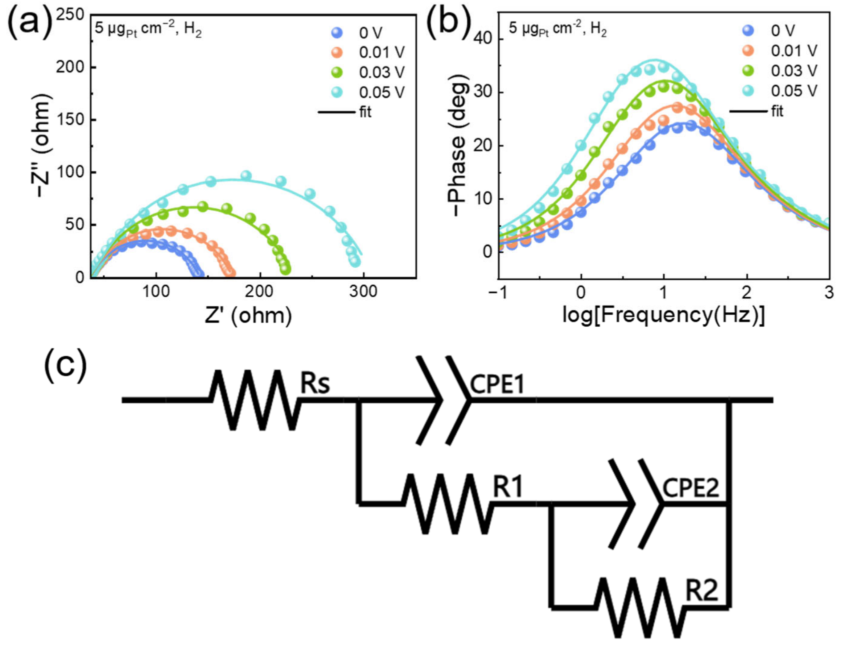

Figure S20. Nyquist plots (a) and corresponding Bode plots (b) of Pt/C recorded at different applied potentials; (c) The fitted equivalent circuit diagram.

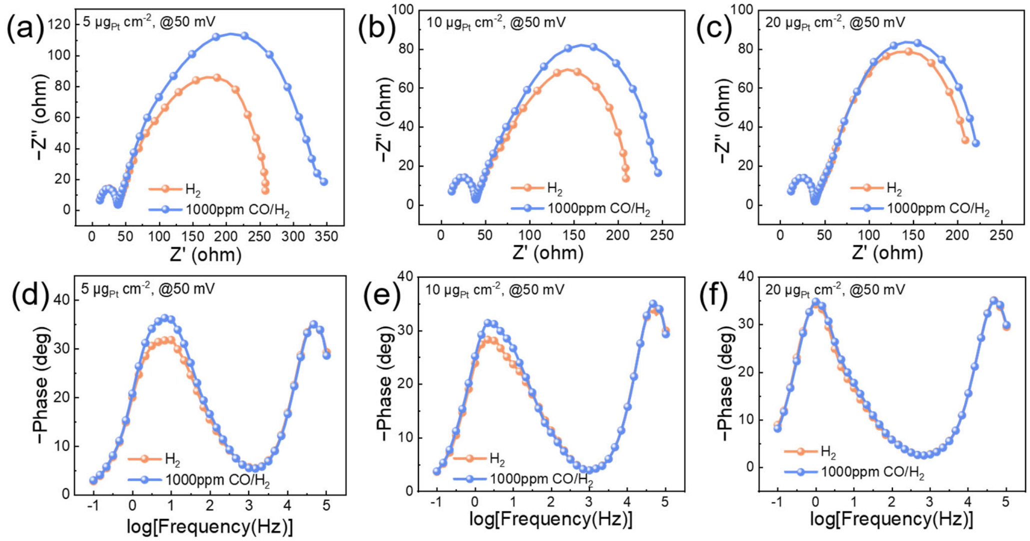

Figure S21. Comparison of Nyquist plots (a–c) and Bode plots (d–f) of Pt/C with catalyst loadings of 5, 10, and 20 μg$_{Pt}$ cm^{-2} at an overpotential of 50 mV in pure H_2 and 1000 ppm CO/H_2 atmosphere.

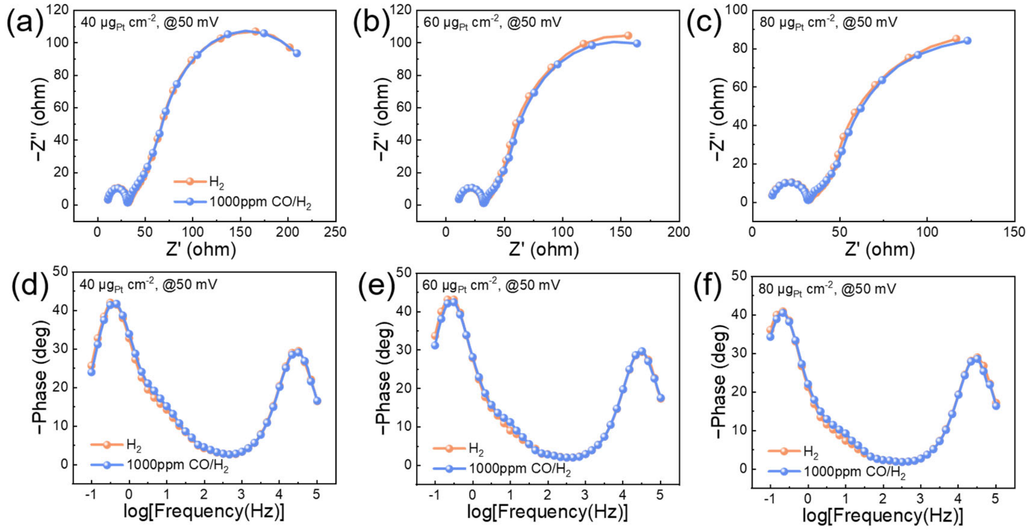

Figure S22. Comparison of Nyquist plots (a–c) and Bode plots (d–f) of Pt/C with catalyst loadings of 40, 60, and 80 μg$_{Pt}$ cm^{-2} at an overpotential of 50 mV in pure H_2 and 1000 ppm CO/H_2 atmosphere.

S3. Supplementary Tables

Table S1. ICP results for the prepared samples.

Sample	Pt Loading
Pt/C)	18.49%

Table S2. EIS fitting results.

Catalyst	η (mV)	R_s (Ω)	CPE1-T (Ω^{-1} s^n)	CPE1-P	R_1 (Ω)	CPE2-T (Ω^{-1} s^n)	CPE2-P	R_2 (Ω)
Pt/C	0	36.84	2.78E-04	0.79632	15.31	3.43E-04	0.70673	90.08
	10	36.95	1.91E-04	0.84233	15.94	4.13E-04	0.70803	120.8
	30	36.78	1.72E-04	0.84844	16.45	3.85E-04	0.72086	179.7
	50	36.2	1.27E-04	0.87431	17.02	4.21E-04	0.73387	255
